# Whole Genome Sequencing and Comparative Genomic Analyses of *Lysinibacillus pakistanensis* LZH-9, a Halotolerant Strain with Excellent COD Removal Capability

**DOI:** 10.3390/microorganisms8050716

**Published:** 2020-05-12

**Authors:** Xueling Wu, Han Zhou, Liangzhi Li, Enhui Wang, Xiangyu Zhou, Yichao Gu, Xiaoyan Wu, Li Shen, Weimin Zeng

**Affiliations:** 1 School of Minerals Processing and Bioengineering, Central South University, Changsha 410083, China; wxlcsu@csu.edu.cn (X.W.); zhhlzl@csu.edu.cn (H.Z.); 185611025@csu.edu.cn (L.L.); weh1204055272@163.com (E.W.); zxy591868@163.com (X.Z.); gyc951122@csu.edu.cn (Y.G.); wuxiaoyan1005@126.com (X.W.); lishen@csu.edu.cn (L.S.); 2 Key Laboratory of Biometallurgy of Ministry of Education, Central South University, Changsha 410083, China

**Keywords:** COD removal capability, halotolerance, *Lysinibacillus* spp., comparative genomics, horizontal gene transfer

## Abstract

Halotolerant microorganisms are promising in bio-treatment of hypersaline industrial wastewater. Four halotolerant bacteria strains were isolated from wastewater treatment plant, of which a strain LZH-9 could grow in the presence of up to 14% (*w*/*v*) NaCl, and it removed 81.9% chemical oxygen demand (COD) at 96 h after optimization. Whole genome sequencing of *Lysinibacillus pakistanensis* LZH-9 and comparative genomic analysis revealed metabolic versatility of different species of *Lysinibacillus*, and abundant genes involved in xenobiotics biodegradation, resistance to toxic compound, and salinity were found in all tested species of *Lysinibacillus*, in which Horizontal Gene Transfer (HGT) contributed to the acquisition of many important properties of *Lysinibacillus* spp. such as toxic compound resistance and osmotic stress resistance as revealed by phylogenetic analyses. Besides, genome wide positive selection analyses revealed seven genes that contained adaptive mutations in *Lysinibacillus* spp., most of which were multifunctional. Further expression assessment with Codon Adaption Index (CAI) also reflected the high metabolic rate of *L. pakistanensis* to digest potential carbon or nitrogen sources in organic contaminants, which was closely linked with efficient COD removal ability of strain LZH-9. The high COD removal efficiency and halotolerance as well as genomic evidences suggested that *L. pakistanensis* LZH-9 was promising in treating hypersaline industrial wastewater.

## 1. Introduction

Hypersaline industrial wastewaters generated from processes, such as food production, petroleum refining, pharmaceutical manufacturing, printing, and dyeing, often contain large amounts of toxic compounds [1,2,3,4,5], most of which were recalcitrant to conventional biological treatment due to inhibition of salt and generally required expensive physico-chemical treatments to remove the salts as well as the organic matter [6]. Under this background, halophilic and halotolerant microorganisms with high chemical oxygen demand (COD) removal efficiency and the capability to convert hazardous compounds to relatively simple compounds, such as H_2_O, CO_2_, CH_4_, and NH_3_ under hypersaline conditions are of increasing interest in industrial wastewater treatment, which is also considered as more environmentally friendly and cost-effective in comparison with physicochemical treatment [7,8].

Members of the genus *Lysinibacillus* belonging to family Bacillaceae. Strains of *Lysinibacillus* were found in diverse environments [9,10,11], and it was reported that some strains of *Lysinibacillus* harbored degradation abilities towards various contaminants [12,13,14,15,16,17,18], and some other strains harbored toxic metal resistance and remove ability, as well as desulfurization and extracellular electron transfer capacity, showing potential for applications in biogeochemical redox processes and waste treatment [19,20,21,22]. Regarding COD removal ability, *Lysinibacillus* sp. RGS applied to decolorize mixture of dyes and actual industrial effluent showed 87% decolorization efficiency with 69% COD reduction at 48 h [23]. These studies emphasized the applicability of *Lysinibacillus* in the treatment of industrial wastewater; however, little research has been conducted to optimize the contaminant removal efficiency as well as explored the salt tolerance limit of *Lysinibacillus* spp. Evolutionary drivers, such as horizontal gene transfer (HGT) and natural selection, may contribute to adaptive evolution of *Lysinibacillus* genomes, whereas their relative contributions were still unexplored.

In this study, we isolated and identified four halotolerant strains from activated sludge of a hypersaline wastewater treatment plant, and we further investigated the COD removal efficiency of the halotolerant strain *L. pakistanensis* LZH-9 under various incubation times, pH, and NaCl concentration. Whole genome sequencing of *L. pakistanensis* LZH-9 and comparative genomic analyses of eight strains of the *Lysinibacillus* from six species was further carried out in order to shed light on the diverse metabolic abilities and stresses resistance of *Lysinibacillus*, and the mechanisms that strains of *Lysinibacillus* employed to degrade organic matter, in an effort to lay the theoretical foundations for detailed characterization of *Lysinibacillus* spp., as well as optimal culturing and scale applications of *Lysinibacillus* species in the wastewater treatment and other bioremediation aspect in the future.

## 2. Materials and Methods

### 2.1. Chemicals, Sampling, and Media

All of the chemicals were purchased from Sinopharm Chemical Reagent Co. Ltd., Shanghai, China, which included tryptone, yeast extract, NaCl, glucose, potassium bichromate, silver sulfate, and sulfuric acid. The activated sludge sample was obtained from the hypersaline pharmaceutical wastewater treatment plant in Changsha, China. The average temperature and pH value of the sample in this study was 21 °C and about 6.2 [24,25]. The latitude and longitude of our sampling location were 28.1355 and 113.0355, respectively. LB medium contained the following (g/L): tryptone, 10; yeast extract, 5; NaCl, 10. The pH of LB medium was adjusted to 7.0–7.2 before autoclaving at 121 °C for 20 min. Synthetic wastewater similar to the concentration of natural sewage was prepared, which was composed of (g/L) glucose, 0.17; soluble starch, 0.16; tryptone, 0.158; beef extract 0.06; KH_2_PO_4_, 0.07; (NH_4_)_2_SO_4_, 0.0284; NH_4_Cl, 0.0255; CH_3_COONa, 0.233; and, Na_2_CO_3_, 0.06. The pH of synthetic wastewater was adjusted to 5.5–6.0 before autoclaving at 115 °C for 30 min. The COD concentration of synthetic wastewater was approximately 800 mg/L.

### 2.2. Enrichment, Isolation and Identification of Halotolerant Bacteria

We suspended 0.1 g above-mentioned activated sludge sample with 10 mL sterile water. Subsequently, one milliliter supernatant was added to the LB medium and the salinity was adjusted to 5% (*w*/*v*) NaCl. The mixed solution was incubated at 30 °C in a shaker at 150 rpm. After three days, 0.5 mL of the solution was inoculated into the LB medium containing 10% (*w*/*v*) NaCl and the other conditions remained unchanged, and then cultured for six days. The above procedures were repeated until the final concentration of NaCl reached 15%. After the enrichment, the solution was serially diluted from 10^−1^ to 10^−9^, and then 0.5 mL of each diluted solution was added to petri dishes and daub uniformly. Four colonies with different morphology were obtained after multiple purifications, and then separately inoculated into LB medium for cultivation. Only the isolate with high COD removal efficiency and salt tolerance was further analyzed. Finally, the isolated strains were identified through 16S rRNA sequence analysis. The bacterial growth under various salt concentrations was observed through the measurement of the absorbance at 600 nm (OD_600_). The sample was examined on a field emission scanning electron microscope (SEM; Nova NanoSEM230, FEI, Hillsboro, OR, USA) after glutaraldehyde solution fixation, phosphate buffer rinsing, gradient ethanol solution dehydration, vacum freeze drying, and gold spray treatment in order to observe microbial morphology.

### 2.3. COD Removal Efficiency Experiments

We studied the removal percentage of COD of *Lysinibacillus pakistanensis* LZH-9 under various culture times (i.e., 1, 2, …, 7, 8 day), initial pH values (i.e., 5.0, 6.0, 7.0, 8.0, 9.0), and salt concentrations (i.e., 0, 1%, 2%, 3%, 4%, 5%) in order to obtain the optimal conditions for *Lysinibacillus pakistanensis* LZH-9 to remove COD. In these experiments, the amount of each bottle of culture solution was 150 mL and the tests were done in triplicate. The method of optimization was referring to previous reports [26,27], and the concentrations of COD were measured by using potassium dichromate method [28]. LZH-9 culture solution was inoculated to LB medium. Subsequently, LZH-9 was cultured for about 24 h until the OD_600_ taking up to 1.0 and the solution was used as seed solution. We inoculated 5% seed solution to the synthetic wastewater and then placed it on a shaker at 30 °C, 150 rpm. The initial pH was 7.0 and the NaCl concentration was 1% during the culture time tests. During the different pH tests, the NaCl concentration was 1%. The final removal efficiencies of COD in pH and NaCl concentration tests were only measured at the 96 h.

### 2.4. DNA Extraction, Genome Sequencing, Assembly and Annotation

Genomic DNA of *Lysinibacillus pakistanensis* LZH-9 was extracted using the Qiagen Genomic DNA Extraction Kit. After the DNA sample quality test was passed, the large fragment was subjected to gelatinization recovery applying BluePippin automatic nucleic acid recovery instrument; the DNA was damaged and repaired; after purification, the DNA fragments were end-repaired and linked with adenine. After purification, the linkers in the kit LSK108 (Oxford Nanopore Technologies, Oxford, United Kingdom) were used to perform the ligation reaction and, finally, qubit [29] was used to accurately quantify the constructed DNA library. After the DNA library was built, a certain concentration and volume of the DNA library was added to a flow cell, and the flow cell was transferred to the Nanopore GridION sequencer for real-time single molecule sequencing (Nextomics Biosciences Institute, Wuhan, China). Cutoffs including mean_qscore_template (>= 7) and sequence length (>= 1000 bp) were applied in order to carry out quality control on the raw data. The reads were first corrected and assembled with the Canu version 1.7 [30]. Pilon version 1.2 [31] was further applied to correct the sequencing errors with default parameters. The corrected genome was tested for circularization by in-house script. Circlator (parameter: fixstart) [32] was used to move the starting point of the sequence to the replication starting site of the genome after removing the redundant parts. After sequencing, the sample yield a total of 1,415,478,110 bp raw data, and the amount of data passed through the quality control was 1,343,227,710 bp. After assembling, correcting, and optimizing, the final genome consisted of a circular chromosome (5,038,663 bp) and a plasmid (66,276 bp) with a total size of 5,104,939 bp. The 16S rRNA sequences of strains LZH-9, LZH-13, LZH-22, and LZH-24 have been deposited at NCBI database under the accession numbers MN121313, MN121312, MN121253, and MN121251, respectively. The whole genome sequence of strain LZH-9 has been deposited at JGI IMG-ER database under the IMG Taxon OID 2823662158 and NCBI database under accession number CP045835-CP045836. *Lysinibacillus pakistanensis* LZH-9 was deposited in China Center for Type Culture Collection (CCTCC) and the accession number was CCTCC AB 2019361.

### 2.5. Average Nucleotide Identity (ANI) and Whole Genome Alignments

Pyani (https://pypi.org/project/pyani/) was used to calculate the average nucleotide identity (ANI) [33] based on Blast algorithm with default parameters. BlastN-based whole genome comparison of strains *L. pakistanensis* LZH-9, *L. pakistanensis* JCM 18776, *L. contaminans* DSM 25560, *L. xylanilyticus* t26, *Lysinibacillus* sp. UBA7518, *L. sphaericus* OT4b.31, *L. mangiferihumi* M-GX18, and *L. parviboronicapiens* VT1065 were performed and represented with BRIG-0.95 [34], and these strains were used as reference, respectively.

### 2.6. Pan-Genome Analyses and Models Extrapolation of Lysinibacillus

Table 2 lists a summary of features for the eight *Lysinibacillus* genomes involved in this study and BUSCO [35] was used to estimate the completeness of each genome against a bacterial core gene set. Gene family clustering followed by genome wide comparisons of eight *Lysinibacillus* representative strains, including *L. pakistanensis* LZH-9, *L. pakistanensis* JCM 18776, *L. contaminans* DSM 25560, *L. xylanilyticus* t26, *Lysinibacillus* sp. UBA7518, *L. sphaericus* OT4b.31, *L. mangiferihumi* M-GX18, and *L. parviboronicapiens* VT1065 together with UniProt search, GO Slim annotation, and GO enrichment analyses (default cutoff *p*-value is 0.05), were performed via OrthoVenn [36] with default parameters. Bacterial Pan Genome Analyses tool (BPGA) pipeline [37] was further used to perform models extrapolation of the *Lysinibacillus* pan/core-genome applying default parameters. The size of *Lysinibacillus* pan-genome was fitted into an power law regression function *Ps* = *κ*n^γ^ with a built-in program of BPGA [37], in which *Ps* was the total number of gene families, *n* was the number of tested strains, and γ stood for free parameters. In case of exponent γ < 0, then the pan-genome of *Lysinibacillus* was suggested to be ‘closed’ because the size of the pan-genome is relatively constant as an additional genome involved. On the contrary, the pan-genome was suggested to be ‘‘open’’ in case of 0 < γ < 1. In addition, the size of the core-genome of *Lysinibacillus* was fitting into an exponential decay function *Fc* = *κ_c_*exp(-*n*/*τ_c_*) with a built-in program of BPGA pipeline [37], in which *Fc* represented the number of core gene families, whereas *κ_c_*, *τ_c_* were free parameters.

### 2.7. Phylogenic Analyses

Phylogenetic tree based on 16S rRNA sequences was constructed with the Neighbor-joining (NJ) method while using MEGA-X [38] with 1000 bootstrap replicates, and phylogenetic trees based on protein sequences of functional genes were constructed using PhyML [39] with the Maximum Likelihood (ML) method and 1000 bootstrap replicates, followed by visualization with iTOL [40], and the sequences were aligned with Muscle [41], and then trimmed with Gblocks [42] while applying a “less stringent” option before tree construction.

### 2.8. Genome Annotation

We applied Rapid Annotation using Subsystem Technology server (RAST) with the “ClassicRAST annotation” scheme [43] and IMG Annotation Pipeline v.5.0.1 [44] as well as KEGG [45] and COG [46] databases for genome annotation. We identified genes that were associated with carbohydrate activity (CAZymes), genes encoding cytochrome P450, and peptidases in *Lysinibacillus* genomes by performing Diamond BlastP [47] against the dbCAN database [48], cytochrome P450 Database [49], and MEROPS database [50], respectively, with cutoff e-value of 1e^−5^, identity 30%, and coverage 70%. Genome neighbor (context) visualization was conducted with EFI-GNT tool [51].

### 2.9. Prediction of Mobile Genetic Elements

We applied the ISFinder [52] to predict and classify insertion sequences (IS) and transposases within *Lysinibacillus* genomes with Blastp (cutoff e-value 1e^−5^). We applied the IslandViewer 4 [53] to detect putative genomic islands (GIs) that were distributed over *Lysinibacillus* genomes. We applied PHASTER (Phage Search Tool Enhanced Release) [54] for detection and annotation of prophage and prophage remnant sequences within *Lysinibacillus* genomes. We also applied CrisprCasFinder [55] for detection of CRISPRs and Cas genes within *Lysinibacillus* genomes. Correlation coefficients (Rs) and two-sided *p* values were obtained by applying Spearman rank correlation analysis (https://www.wessa.net/rwasp_spearman.wasp/).

### 2.10. Genome-wide Detection of Positively Selected Genes and Codon Adaption Index (CAI) Calculation

Comparisons of non-synonymous (dN) to synonymous (dS) substitution rate (as ω = dN/dS) have been widely applied in order to figure out whether the mutations that change the amino acid (dN) in a specific position are adaptive (ω > 1, positive selection), deleterious (ω < 1, negative selection) or neutral (ω = 1, neutral evolution), and we used PosiGene pipeline [56] for genome-wide detection of positively selected genes in above-mentioned genomes of *Lysinibacillus* spp. (Table 2), in which *L. pakistanensis* LZH-9 was used as the anchor, reference, and target species. Genes were considered to be PSGs if the branch-wide test resulted in a false discovery rate (FDR) of <0.05 and an adjusted *p* value of <0.05. We used CAI as a numerical estimator of gene expression level. We used CAIcal [57] in order to calculate CAI values of genes of above-mentioned strains. PCoA based on Bray-Curtis distance was performed with Origin Pro 2017 software (OriginLab, Northampton, MA, USA)

## 3. Results and Discussion

### 3.1. Isolation and Identification of Halotolerant Bacteria

**Figure 1:**
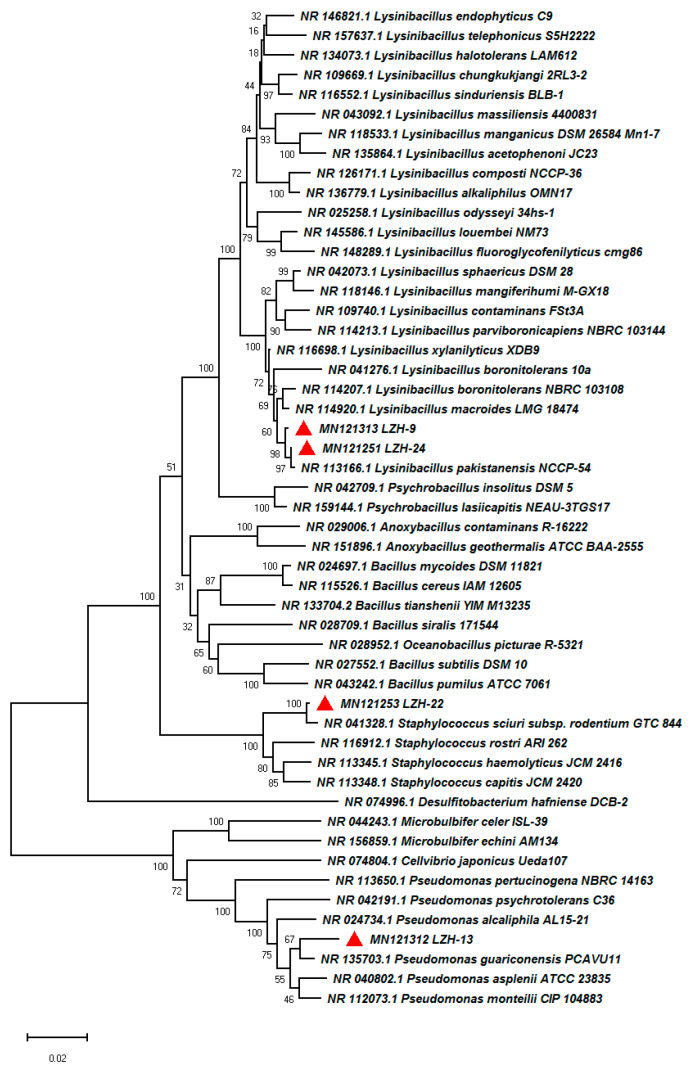
Phylogenetic tree based on 16S rRNA sequences with the Neighbor-joining (NJ) method of LZH-9, LZH-13, LZH-22, LZH-24, and other representative species. Bootstrap values are indicated at each node based on a total of 1000 bootstrap replicates.

**Figure 2:**
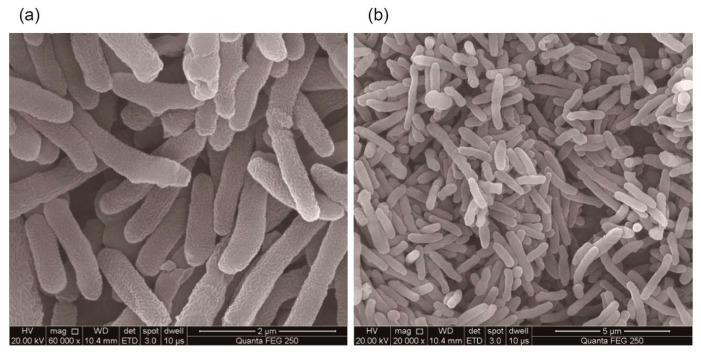
Scanning electron micrographs of strain *Lysinibacillus pakistanensis* LZH-9: (**a**) with magnification of 60,000; and (**b**) with magnification of 20,000.

**Figure 3:**
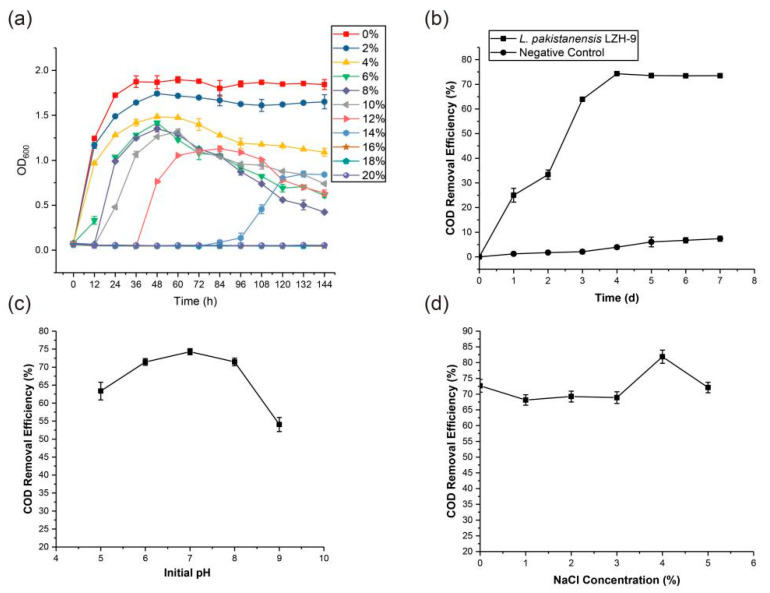
(**a**) Growth curves of strain *Lysinibacillus pakistanensis* LZH-9 under different NaCl concentrations, tested in triplicate. (**b**) Effects of incubation time on the COD removal efficiency percentage in synthetic wastewater. The initial concentration of NaCl was 1%, inoculum concentration was 5% and strain LZH-9 was cultured with an initial pH 7.0. (**c**) Effects of initial pH on the COD removal efficiency percentage in synthetic wastewater. The effect of initial pH was tested at the 4th day. The initial concentration of NaCl was 1%. (**d**) The effects of initial NaCl concentration on the COD removal efficiency in synthetic wastewater. The effect of initial NaCl concentration was tested at the 96 h.

After enrichment, isolation, and purification, four strains LZH-9, LZH-13, LZH-22, and LZH-24 were obtained and phylogenetic analysis based on 16S rRNA sequences was conducted (Figure 1). All of the strains were capable of removing COD, and strain LZH-9 was selected for further optimization and analyses, since it possessed the highest COD removal efficiency 69.8% (Table 1). The colony of strain LZH-9 on LB (Lysogeny broth) solid medium was light yellow, smooth, moist, with neat edges, and as seen under a scanning electron microscope, strain LZH-9 has rod shape and folded surface with a size of ~0.4 μm × (1.5–3) μm (Figure 2). Notably, strain LZH-9 could grow in the presence of up to 14% (*w/v*) NaCl (Figure 3a).

### 3.2. Optimization of COD Removal Efficiency with Strain LZH-9

The removal efficiency of COD increased largely during the first 96 h and it reached 74.3% at 96 h (Figure 3b). With the pH increasing from 5.0 to 7.0, the removal of COD consistently increased and reached the maximum value at pH 7.0 (Figure 3c), whereas, with initial pH increasing from 7.0 to 9.0, the COD removal percentage decreased from 74.3% to 54.1%. It was concluded that with increasing of initial pH, COD removal percentage showed a trend of first increase, followed by decrease.

Osmotic pressure that is caused by saline concentrations (>1% salt) would cause plasmolysis or loss of biological activity in microbes [58]. It was reported that the COD removal efficiency of wastewater with rotating biological disc system fell from 85% to 59% when salinity increased from 0 to 5%, and the COD removal efficiency would decrease to below 80% when the salt concentration was over 50 g/L in Fed-Batch Operation [58,59]. In this study, we found that strain LZH-9 had a maximal salt tolerance of 14% (*w*/*v*) NaCl, higher than that of *L. halotolerans* LAM612^T^, which could resist up to 10% (*w*/*v*) NaCl [18], and LZH-9 presented the highest salt tolerance amongst reported strains of genus *Lysinibacillus* thus far. Of synthetic wastewater with NaCl concentration lower than 5%, the COD removal efficiency varied from 68.1% to 81.9% at 96 h (Figure 3d), whereas with a NaCl concentration higher than 5%, the COD removal efficiency fell below 60.0%. These results suggested that the strain LZH-9 could remove COD in high salt conditions (1%–5% NaCl). Kubo at el. [3] isolated two salt-tolerant bacteria (resisted up to 15% NaCl), in a mixed culture with both strains the COD removal efficiency was approximately 70% for 72 h in a flask, and it increased to about 90% when they were applied in a pilot plant (working volume 1m^3^) for 7 d. Mehdi Ahmadi at el. [2] isolated three salt-tolerant bacteria and observed that the COD removal efficiency was 78.7%–61.5% in the treatment of real saline wastewater with a decreasing trend along with increasing of the organic loading rate. Comparatively, strain LZH-9 was among the most efficient COD removal bacteria, illustrating notable potential as a microorganism resource for the bio-treatment of hypersaline wastewater.

### 3.3. Genomic Features

The genome of LZH-9 consisted of a circular chromosome (5,038,663 bp) and a plasmid (66,276 bp) with a total size of 5,104,939 bp. A total of 5263 CDS including 108 tRNA and 34 rRNA were predicted in the complete genome of strain LZH-9 while using IMG Annotation Pipeline v.5.0.1 [44]. Whole genome BLASTN-based average nucleotide identity (ANI) analyses showed that strain LZH-9 had ANI values that were above the cutoff (96%) with strains *L. pakistanensis* JCM 18,776 (99.0%) and *Lysinibacillus* sp. UBA7518 (96.8%) (Figure S1), thus these three strains were classified into the specie *L. pakistanensis*. We also chose five other genome of *Lysinibacillus* spp. that were phylogenetically close with strain LZH-9 from public database, and a summary of features for the eight *Lysinibacillus* genomes involved in comparative study were listed in Table 2. The G+C contents of the 8 genomes ranged from 36.3% to 37.8%. These genomes varied in coding density from 71.5% to 83.7%. In addition, whole genome comparison of *Lysinibacillus* spp. while using BLAST Ring Image Generator (BRIG) revealed that genome of strain LZH-9 was rather conserved amongst the species *L. pakistanensis* showing high similarity, whereas many short non-common-shared genomic regions were also found in each *Lysinibacillus* genome, most of which harbored poorly characterized proteins (Figures S2 and S3).

### 3.4. Core and Pan-genome of Lysinibacillus

**Figure 4:**
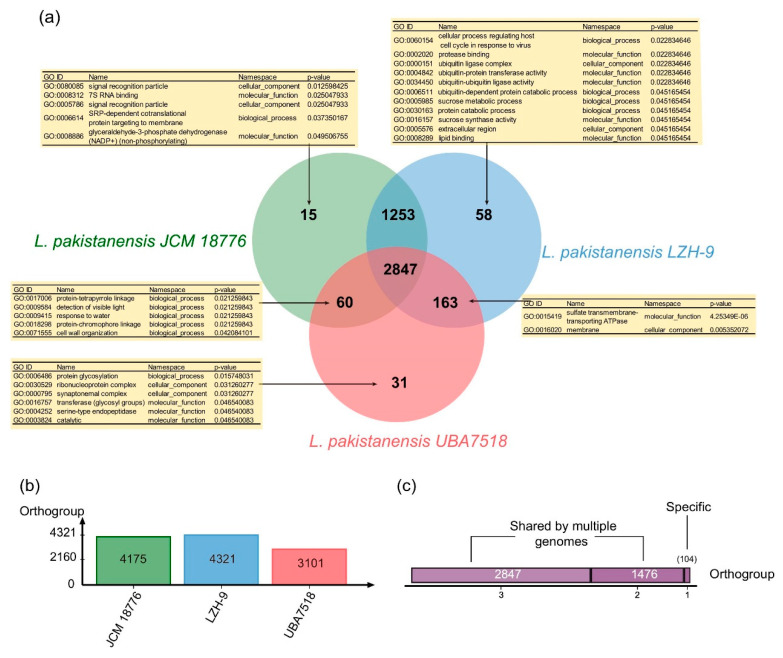
(**a**) Venn diagram showing the numbers of orthogroup including the core genome (shared by three strains of *L. pakistanensis*), the accessory genome (shared by some but not at all of these strains) and strain-specific gene families in individual genomes; enriched Gene ontology (GO) terms (*p* < 0.05) in respective orthogroups are shown in the tables. (**b**) Bar chart showing the total numbers of orthogroup identified in each genome of the three strains of *L. pakistanensis*. (**c**) The total numbers of orthogroups of strain-specific gene families (=1) or the accessory genome (shared by two, three strains) and core genome of three strains of *L. pakistanensis*.

The pan-genome three strains of *L. pakistanensis* possessed 4427 gene families, whereas core genome possessed 2847 gene families accounting for 64.3% of all gene families (Figure 4). Clusters of Orthologous Groups (COG) annotation of the pan-genome of three *L. pakistanensis* strains revealed that the core genome had a higher proportion of genes in COG [J] [F] [O] [U] [E] [D] [C] [H] and [N] associating with basic biological function linking with ribosome, nucleotide, translation, amino acid, division, energy, and motility in comparison with accessory genome and strain-specific genes, whereas the accessory genome had a higher proportion of genes related to COG [T] [K] [V] [I] associating with transcription, signal transduction, defense, and lipid metabolism, and strain-specific genes had a higher proportion of genes that were categorized into COG [L], [M], [P], and [G] and [Q] (Figure S4). In addition, Gene Ontology (GO) enrichment analyses showed that functions significantly enriched (*p*-value < 0.05) in strain-specific gene families of *L. pakistanensis* LZH-9 were mostly related to metabolic process of sucrose and protein (Figure 4).

**Figure 5:**
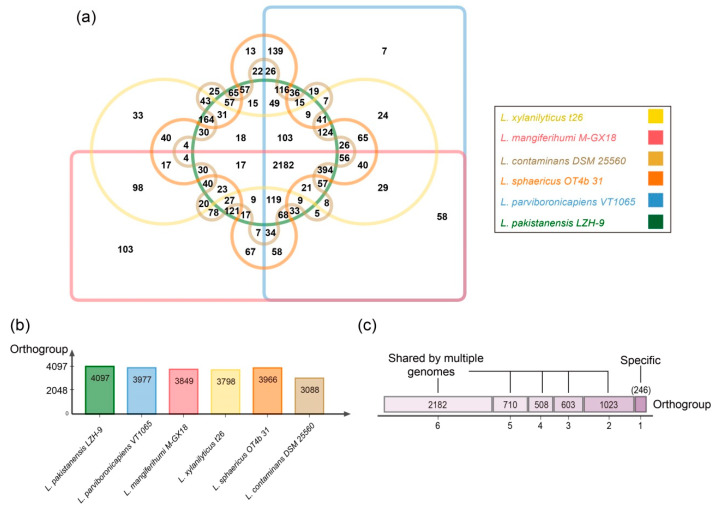
(**a**) Venn diagram showing the numbers of orthogroup including the core genome (shared by six representative strains of *Lysinibacillus*), the accessory genome (shared by some but not at all of these strains) and strain-specific gene families in individual genomes; enriched Gene ontology (GO) terms (*p* < 0.05) in respective orthogroups are shown in the tables. (**b**) Bar chart showing the total numbers of orthogroup identified in each genome of six representative strains of *Lysinibacillus*. (**c**) The total numbers of orthogroups of strain-specific gene families (=1) or the accessory genome (shared by two, three, four, five, six strains) and core genome of six representative strains of *Lysinibacillus*.

The pan-genome analyses of six strains from six different *Lysinibacillus* species showed that 2182 (41.4%) out of total 5272 genes families were shared by all tested strains (Figure 5). Additionally, *L. pakistanensis* LZH-9 had the most genes families (4097) in its genome. Mathematical modeling revealed a ‘‘open’’ pan genome fitted into a power law regression function [*P_s_* (*n*) = 4276.74*n*^0.444721^] with a parameter (γ) of 0.444721 falling into the range 0 < γ < 1, whereas core genome was fitted into an exponential regression [*F_c_* (*n*) = 4475.34*e*^−0.177936n^] (Figure S5). COG annotation showed that core genome had a higher proportion of genes that were involved in COG [J] [F] [O] [U] [E] [D] [C] [H] and [I], associating with central biological function than accessory genome and unique genes, whereas the accessory genome contained a higher proportion of genes related to COG [T] [P], and the unique genes had a higher proportion of genes categorized into COG [V] [Q] [M] [K] [L] [G] (Figure S6). GO enrichment analyses showed that the only GO term significantly enriched (*p*-value < 0.05) in core genome was glycolytic process (Table S1), reflecting the considerable catabolic capability of carbohydrate of *Lysinibacillus*. Additionally, functions significantly enriched (*p*-value < 0.05) in accessory genome and strain-specific gene families were mostly related to substrate transport, signal transduction and regulation, catabolic process of various carbon source, diverse nitrogen source, metabolic process of antibiotics and other toxic compounds, which in a way reflected that these *Lysinibacillus* strains harbored potential for biodegradation application, in consideration of the above-mentioned enriched catabolism related pathways.

### 3.5. Functional Potential and Phylogenetic Analyses

#### 3.5.1. Carbon Metabolism

**Figure 6:**
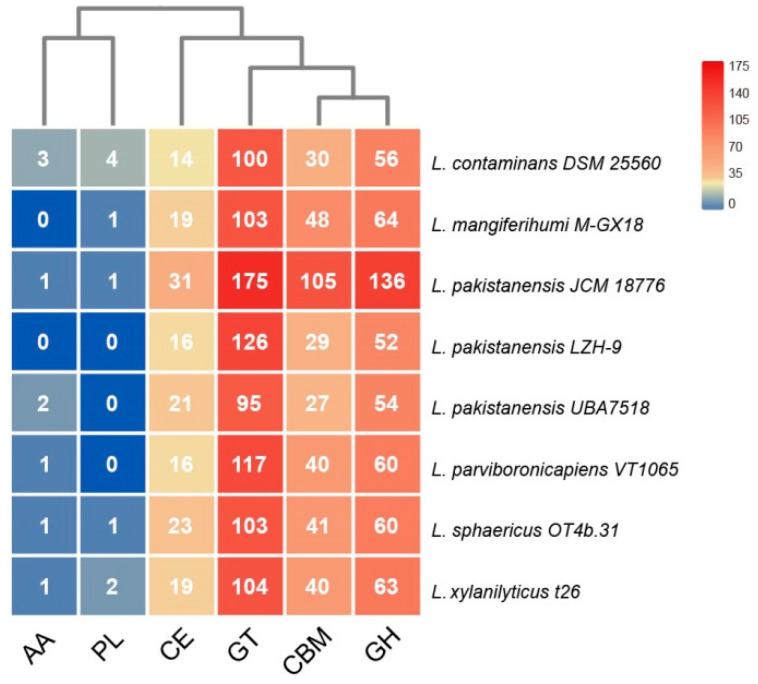
Heat map showing the distribution and numbers of genes encoding carbohydrate active enzymes (CAZymes) in eight different *Lysinibacillus* strains in this study. Abbreviations in the chart: AA, auxiliary activities; CBM, carbohydrate-binding molecules; CE, carbohydrate esterases; GH, glycoside hydrolases; GT, glycosyltransferases; PL, polysaccharide lyases.

Central carbohydrate metabolism including the glycolysis and gluconeogenesis, oxidative tricarboxylic acid cycle (TCA), pentose phosphate pathway (PPP), glyoxylate bypass, acetogenesis, methylglyoxal metabolism, and genes involved in metabolism of organic acids glycerate and lactate were found in all tested genomes of *Lysinibacillus*. Genes that were involved in biosynthesis of butanol, butyrate, acetolactate, metabolism of acetoin, butanediol, glycerol, and utilization of chitin and N-acetylglucosamine were also present in all tested genomes, and genes related to ethanolamine metabolism were present in all of the tested genomes, except for *L. contaminans* DSM 25560. Genes that were related to metabolism of monosaccharides, such as D-ribose, deoxyribose, and deoxynucleoside, D-gluconate and ketogluconates were also detected in all of the tested genomes, and genes related to mannose metabolism were present in all tested genomes except for *L. xylanilyticus* t26. Phylogenetic analyses indicated that genes encoding mannose-6-phosphate isomerase, gluconate 2-dehydrogenase, ribokinase of *Lysinibacillus* spp. involved in the metabolism of mannose, D-gluconate, and ketogluconates, and PPP, respectively, were likely acquired via cross-family gene exchange or HGT events from Planococcaceae or Paenibacillaceae, and genes encoding glycerophosphodiester phosphodiesterase of *Lysinibacillus* involved in glycerol metabolism were likely acquired via cross-order HGT from Lactobacillales (Figures S7–S10). Annotation against dbCAN (database of carbohydrate-active enzyme) [48] also revealed that *Lysinibacillus* spp. harbored an abundant repertoire of carbohydrate active enzymes (CAZymes), including carbohydrate esterases (CEs), carbohydrate binding molecules (CBMs), glycosyltransferases (GTs), glycoside hydrolases (GHs), auxiliary activities (AAs), and a small number of polysaccharide lyases (PLs), of which GTs were most abundant, and strain *L. pakistanensis* JCM 18,776 possessed the most carbohydrate active enzymes (Figure 6). All of these enzymes involved in carbon metabolism are closely linked with the COD removal ability of *Lysinibacillus*, through which carbohydrate-containing contaminants can be consumed by microbes while supplying energy for microbes at the same time.

#### 3.5.2. Nitrogen and Sulfur Metabolism

Nitrogen- and sulfur-containing contaminants also contribute to COD concentrations. We found in all tested genomes of *Lysinibacillus* genes encoding nitric oxide synthases that helped to oxidize L-arginine to nitric oxide (NO), which might protect the bacteria against oxidative stress [61,62], and nitric oxide dioxygenases encoded by *hmp* genes that oxidized nitric oxide to nitrate. Genes encoding nitrilase that catabolized organic nitrogen sources to produce ammonia were found in the genomes of *L. contaminans* DSM 25,560 and *L. sphaericus* OT4b.31. Gene clusters encoding urease composed of the functional subunits (*ureAB* and *ureC*) and accessory proteins (*ureD*, *ureE*, *ureF*, and *ureG*) that converted urea into molecule ammonia and carbon dioxide [63] were only found in *L. parviboronicapiens* VT1065 and *L. sphaericus* OT4b.31. Additionally, genes encoding ammonium transporter, glutamate dehydrogenase, glutamine synthetase, and carbamoyl-phosphate synthase were found in all of the tested genomes of *Lysinibacillus*, through which enzymes a series of important biosynthesis reactions were carried out with ammonia as entry. However, denitrifying reductase genes were missing in all the tested genomes of *Lysinibacillus*. Genes encoding sulfate and thiosulfate permease, sulfate adenylyltransferase, adenylyl-sulfate reductase, phosphoadenylyl-sulfate reductase, and assimilatory sulfite reductase involved in reversible assimilatory sulfate reduction or indirect sulfite oxidation were also found in all the tested genomes of *Lysinibacillus*. It seemed that utilizations of ammonia, organic nitrogen sources, and sulfate for growth were the main strategies of tested *Lysinibacillus* spp.

#### 3.5.3. Energy Conservation and Transduction

All of the tested genomes of *Lysinibacillus* contained gene clusters *qcrABC* involved in the synthesis of menaquinone cytochrome c reductase complexes functioned preferentially under anaerobic to microaerobic conditions, which coupled the transfer of electrons from quinol in the membrane to c-type cytochrome [64,65]. The succinate:quinone oxidoreductase (complex II) that linked the TCA cycle to the quinone pool [66], cytochrome c oxidases (complex IV) that transferred electrons from cytochrome c to oxygen [67], and oxygen-reducing bd-type oxidase encoded by *cydAB* genes were also present in all of the tested genomes. In addition, genes encoding NADH:quinone oxidoreductase (complex I) were not detected.

### 3.6. Environmental Adaption

#### 3.6.1. Resistance to Antibiotics and Toxic Metals

Genes that were involved in vancomycin resistance, such as *vanW* encoding vancomycin B-type resistance proteins, were present in all tested genomes of *Lysinibacillus*, acquired likely via cross-family HGT events (Figure S11), whereas *vanRS* encoding related two-component signal transduction systems [68,69] were found in all the tested genomes of *Lysinibacillus*, except for *L. contaminans*, and *L. mangiferihumi*, acquired likely via cross-class HGT events from members of Clostridiales (Figures S12 and S13). Genes *fosB* that encoding fosfomycin resistance protein [70], tetracycline resistance genes *tet(M)* and *tet(O)* encoding paralogs of the translational GTPase, the elongation factor EF-G were present in all of the tested genomes, through which tetracycline was actively removed from the ribosome of bacteria [71,72], and phylogenetic analyses suggested that *fosB* genes of *Lysinibacillus* were likely acquired via cross-family gene exchange or HGT events (Figure S14). Genes encoding aminoglycoside adenylyltransferases that adenylated streptomycin and spectinomycin [73] were present in all of the tested genomes, except for *L. contaminans* DSM 25,560 and *L. sphaericus* OT4b.31, whereas genes *satA* encoding N-acetyltransferases that inactivated streptothricin via acetyl-CoA-dependent lysine acetylation [74,75] were only found in strains of *L. pakistanensis* and strain *L. xylanilyticus* t26, likely acquired via cross-order HGT (Figure S15). Genes encoding beta-lactamases involved in resistance to beta-lactam antibiotics were present in all tested genomes, except for *L. contaminans* DSM 25560, acquired probably via cross-class HGT from members of Clostridiales or Tissierellales (Figure S16). Gene clusters *bceRSAB* and *yvcSRQP* encoding bacitracin export systems that are involved in responses and resistance to bacitracin [76,77,78] and genes *cbrC* encoding colicin E2 tolerance protein [79] were only present in the strains of *L. pakistanensis* and strains *L. xylanilyticus* t26, *L. sphaericus* OT4b.31, which were probably acquired via cross-order HGT from Clostridiales (Figure S17). As for resistance to heavy metals, we found that genes *chrA* encoding chromate transport proteins were present in all of the tested genomes, except for *L. contaminans* DSM 25560. Arsenic resistance genes *arsC* encoding arsenate reductases that converted arsenate to arsenite and *arsM* encoding arsenic methyltransferases that converted inorganic arsenic into volatile derivatives were present in all the tested genomes of *Lysinibacillus*. Arsenite can then be expelled from the cells by arsenite efflux pump encoded by *acr3* or *arsB* of *Lysinibacillus* spp., which were probably acquired via cross-family HGT events (Figures S18–S20), and *acr3* were present in all tested genomes of *Lysinibacillus*. Gene clusters which involved in efflux of divalent heavy metal cations including *cadAC* that encoded putative ATP-dependent efflux systems and genes *czcD* encoding cation diffusion facilitator (CDF) proteins were also present in all of the tested genomes of *Lysinibacillus*, both of which were acquired likely via cross-family HGT events (Figures S21–S23) [80,81]. Genes *copA* encoding copper-translocating P-type ATPase that transported Cu(I) ions from the cytosol to the periplasm were also present in all tested genomes. All of these genes may confer *Lysinibacillus* spp. resistance to toxic compound, such as antibiotics and heavy metal ions in polluted water, or other environmental contaminants, enhancing their environmental adaptivity and bioremediation ability.

#### 3.6.2. Capsular and Extracellular Polysacchrides

Capsular and extracellular polysacchrides (EPS) play important roles in cell adhesion and biofilm formation, which is closely related to the colonization, biodegradation, desiccation, and toxic compound resistance of bacteria [82,83,84,85]. Gene clusters *rfbABCD-wbbL* encoding proteins that converted glucose-1-phosphate to the EPS precursor d-TDP-rhamnose were detected in the strains *L. pakistanensis* JCM 18,776 and *L. contaminans* DSM 25,560 [86,87], whereas gene clusters *epsBCD* and *epsEF* that were also involved in biosynthesis of EPS [88,89] were detected in all of the tested genomes, except for those of *L. pakistanensis*. In addition, genes *hasA* encoding hyaluronan synthases and *hasC* encoding UTP-glucose-1-phosphate uridylyltransferase involved in the biosynthesis of hyaluronic acid capsule [90] were found in strains *L. mangiferihumi* M-GX18, *L. parviboronicapiens* VT1065 and strains of *L. pakistanensis*. Genes *pda* encoding polysaccharide deacetylases and genes *pgd* encoding peptidoglycan N-acetylglucosamine deacetylase mediating peptidoglycan deacetylation in protection against lysozyme [91,92,93] were present in all of the tested genomes. We also found *luxS* genes encoding S-ribosylhomocysteinase present in all tested genomes, acquired via cross-family gene exchange or HGT events (Figure S24). Autoinducer-2 (AI-2) produced by the S-ribosylhomocysteinase (LuxS), formed a universal quorum sensing system that facilitated both inter- and intra-genomes communication and played important roles in growth regulation, EPS production, and biofilm formation [94,95,96,97], and induction of gene *luxS* under salt and chloride stress was also observed in a Bacillaceae member [98].

#### 3.6.3. Halotolerance and Resistance to Osmotic Stress

Biological processes, including sodium efflux, potassium uptake, and compatible solute uptake and synthesis, are known to counteract osmotic stress [99]. Gene clusters *kdpEDABC* that enhanced resilience to salt stress by scavenging K^+^ [100,101,102,103], and genes *kefA* encoding mechanosensitive channel family proteins that regulated ion homeostasis and turgor pressure of bacteria upon growth at high osmolarity [104,105] were present in all of the tested strains except for *L. contaminans* DSM 25560, and phylogenetic analyses revealed that *kdp* gene clusters were clustering with those from Clostridiales and Planococcaceae, indicating that gene clusters *kdp* were likely acquired via HGT (Figures S25–S28). Another ATP-dependent transporters of monovalent cation (K^+^ and Na^+^) present in all of the tested strains contributed to salt resistance were KtrAB, composed of cytosolic octameric regulatory proteins (KtrA) and dimeric membrane proteins (KtrB) [100,106,107], acquired likely via cross-family gene exchanges or HGT events (Figure S29). The accumulation of small, uncharged compatible solute, such as glycine betaine in the cytoplasm, is also a common strategy of bacteria to counteract external salt stress, and previous reports also illustrated the possible additional functions of glycine betaine as cold and heat stress protectant [108,109,110]. Most bacteria uptake glycine betaine with different transporters or synthesize glycine betaine from choline. Choline dehydrogenase (BetA) induced by salt and/or choline, together with glycine betaine aldehyde dehydrogenase (BetB), catalyzed the two-step oxidation of choline to glycine betaine [111], and we found genes *betA* present in *L. xylanilyticus* t26 and strains of *L. pakistanensis* and *betB* present in *L. xylanilyticus* t26 and *L. contaminans* DSM 25560. Phylogenetic analyses showed that the genes *betA* of *Lysinibacillus* were clustering with those from Paenibacillaceae and Thermoactinomycetaceae suggesting cross-family gene exchanges (Figure S30). The OpuA system is the main ATP-binding cassette (ABC) transporter for glycine betaine consisting of three components: a hydrophilic polypeptide encoded by *opuAC*, which is a glycine betaine-binding protein (GBBP), an integral membrane protein encoded by *opuAB*, and an ATPase encoded by *opuAA* [110,112,113]. We found that genes *opuAA*, *opuAB*, and *opuAC* were present in all of the tested strains, being probably acquired from members of Lactobacillales via cross-order HGT (Figures S31–S33). We also found gene clusters *proXV* involved in transport of glycine betaine present in all tested strains [114,115]. In addition, genes *proCBA* that were involved in biosynthesis of proline, an effective osmolyte important in salt tolerance [116,117,118] were also present in all tested genomes.

#### 3.6.4. Xenobiotics Biodegradation and Metabolism

KEGG (Kyoto Encyclopedia of Genes and Genomes) annotation revealed multiple genes involved in xenobiotics biodegradation and metabolism that closely correlated with contaminant removal ability in the tested genomes of *Lysinibacillus* spp. As for genes associated with benzoate degradation, genes encoding 3-hydroxybutyryl-CoA dehydrogenase (*fadB*), 4-oxalocrotonate tautomerase (*xylH*), acetyl-CoA C-acetyltransferase (*atoB*), acetyl-CoA acyltransferase (*fadA*), and catechol 2,3-dioxygenase (*dmpB*) were found in all of the tested genomes of *Lysinibacillus*, of which *xylH* was essential in the conversion of many aromatic compounds to intermediates of the TCA cycle [119], and the *atoB* and *fadB* genes were likely acquired via cross-family HGT events (Figures S34 and S35), and genes encoding 2-keto-4-pentenoate hydratase (*mhpD*), 2-oxo-3-hexenedioate decarboxylase (*dmpH*), and aminomuconate-semialdehyde/2-hydroxymuconate-6-semialdehyde dehydrogenase (*dmpC*) were only present in strains of *L. pakistanensis*. Genes encoding 3-hydroxyacyl-CoA dehydrogenase/enoyl-CoA hydratase (*fadJ*), and 4-carboxymuconolactone decarboxylase (*pcaC*) were missing in strain *L. sphaericus* OT4b.31, and *fadJ* genes were likely acquired via cross-class HGT events from members of Burkholderiales or Clostridiales (Figure S36). As for chloroalkene degradation, genes encoding 2-haloacid dehalogenase, alcohol dehydrogenase (adh), aldehyde dehydrogenase (NAD^+^) (*aldh*) were also present in all of the tested genomes of *Lysinibacillus*. Additionally, genes encoding 4-hydroxy 2-oxovalerate aldolase (*mhpE*), acetaldehyde dehydrogenase (*mhpF*) involved in xylene and dioxin degradation were only present in strains of *L. pakistanensis*. As for genes that associated with aminobenzoate degradation, genes encoding acylphosphatase and 4-nitrophenyl phosphatase that likely acquired via cross-family HGT events (Figure S37) were present in all the tested genomes of *Lysinibacillus*. As for aromatic amin catabolism, genes encoding 4-hydroxyphenylacetate 3-monooxygenase (*hpaB*) and nitrilotriacetate monooxygenase (*ntaB/nmoB*) that likely acquired via cross-family HGT events (Figure S38) were present in all of the tested genomes of *Lysinibacillus*. Gene clusters *dmpR-hpaB-dmpB-pnpA-mhpD-dmpFGH-xylH-hpaE* with identical arrangement involved in xenobiotics biodegradation and metabolism were present in genomes of *Lysinibacillus*, whose non-uniform gene contexts and deviant GC contents from that of genomes suggested that it was likely acquired via HGT after the speciation of *Lysinibacillus* (Figure S39). Annotation against the MEROPS database [50] showed numbers of peptidases that helped to hydrolyze proteinous contaminants in the genomes of *Lysinibacillus*, of which strain JCM 18,776 contained the most genes encoding peptidases (287), followed by strain LZH-9 (159), and both strains belonged to specie *L. pakistanensis*. Cytochrome P450 represent a super family of heme-containing monooxygenases that played critical roles in the adaptation of microbes to diverse environments by modifying harmful environmental chemicals, and annotation against cytochrome P450 Database [49] revealed that strain *L. pakistanensis* JCM 18,776 contained the most genes encoding cytochrome P450 (66), followed by strain *L. xylanilyticus* t26 (44) and strain *L. pakistanensis* LZH-9 (43) (Table S2).

#### 3.6.5. Mobile Genetic Elements and CRISPR-Cas Systems

Mobile genetic elements (MGEs) are moveable genome segments, such as insertion sequences, transposases, genomic islands (GIs), and phages, and the amount of MGEs positively correlates with the frequency of HGT [120]. Results showed that an abundant repertoire of MGEs as well as CRISPR-Cas (clustered, regularly, interspaced, short, palindromic repeats -associated genes) systems existed in *Lysinibacillus* genomes. The number and the total length of transposon sequences per genome can reach 158 and 20.9 kb (*L. pakistanensis* LZH-9). The number and the total length of GIs related sequences per genome can reach 621 and 317.8 kb (*L. mangiferihumi* M-GX18), and the number and the total length of phage related sequences can reach 550 and 430.8 kb (*L. mangiferihumi* M-GX18) (Table S3). On the other hand, CRISPR-Cas systems are immune system of prokaryote against viral attack [121], and Type I-B CRISPR-Cas systems were founded in the tested genomes, in which *L. pakistanensis* LZH-9 contained the most (76) CRISPR-Cas related genes or spacers (Table S3). We found in the tested genomes that the numbers of identified transposons sequences correlated positively (*rho* = 0.881, *p* = 0.007) with the total length of transposons sequences, the same as the numbers of phage sequences with total length of phage region (*rho* = 0.922, *p* = 0.001) and the number of CRISPR-Cas sequences and total length of CRISPR-Cas sequences (*rho* = 0.878, *p* = 0.004). However, the number of genomic island sequences in tested genomes did not significantly correlate with the length of genomic island (*rho* = 0.476, *p* = 0.243) (Figure S40). The abundant MGEs present in tested genomes of *Lysinibacillus* indicated that HGT may contribute to the genomic evolution of *Lysinibacillus* genomes during niche adaption, and CRISPR-Cas system may also help to protect the genomes of *Lysinibacillus* and eliminate harmful genomic intrusions.

### 3.7. Positive Selection Analyses

Positive selection was also found to be an important driving force for evolution of *Lysinibacillus*. Genes can be changed by positive selection for fixation of beneficial gene variants in a population/specie over time if they increased fitness. Genome-scale positive selection analyses were performed on eight genomes of *Lysinibacillus* in this study (Table 2). Seven genes (Lp_411, Lp_718, Lp_1054, Lp_2135, Lp_3474, Lp_3540, Lp_4098) were identified as being under positive selection (Table S4), two (Lp_2135, Lp_3540) of which were annotated as hypothetical proteins. Gene Lp_1054 encoding an uncharacterized conserved protein was located in a known gene cluster related to flagellum biosythesis. Gene *thiJ* (Lp_411) encoded protein performing multiple functions including protease/amidase activity of broad specificity, acid resistance, oxidative stress resistance and holdase chaperone activity [122,123,124,125] and gene *surA* (Lp_4098) encoded a protein functioned as periplasmic chaperone and peptidyl-prolyl isomerase (PPIase) that is involved in cell envelope functions, biogenesis of β-barrel outer membrane proteins (OMPs) and virulence mediation [126,127,128,129], both of which may play important roles in maintaining cellular environment homeostasis. Gene *fabB* (Lp_718) encoding β-ketoacyl-ACP synthase capable of catalyzing the elongation of longer-chain-ACPs during fatty acid synthesis [130,131,132], and gene *trpF* (Lp_3474) encoding phosphoribosylanthranilate isomerase involved in tryptophan synthesis were also found containing adaptive changes. Taken together, those genes under positive selection in tested *Lysinibacillus* genomes were prone to playing a variety of functional roles with broad substrate specificity, of which even small adaptive changes in their coding sequences may bring considerable benefits in evolution.

### 3.8. Expression Assessment with Codon Adaption Index (CAI)

**Figure 7:**
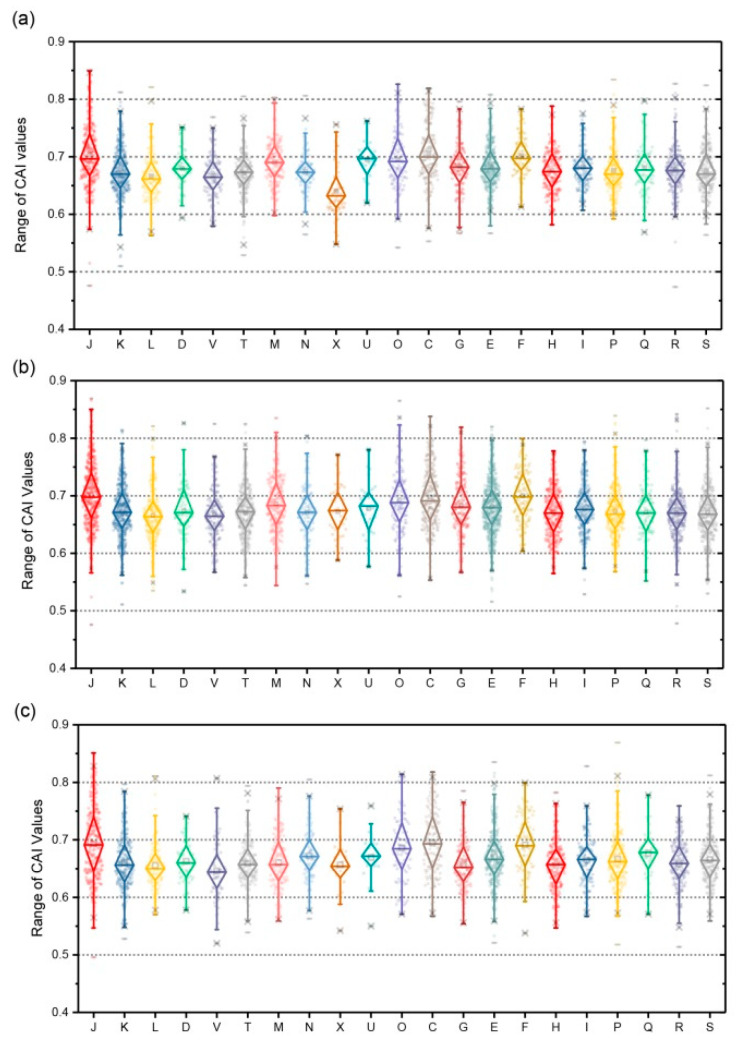
(**a**) Ranges of Codon Adaption Index (CAI) values of different genes in the genome of *L. pakistanensis* LZH-9 based on COG classification. (**b**) Ranges of CAI values of different genes in the genome of *L. pakistanensis* JCM 18,776 based on COG classification. (**c**) Ranges of CAI values of different genes in the genome of *Lysinibacillus* sp. UBA7518 based on COG classification. (COG categories description J: Translation, ribosomal structure and biogenesis; K: Transcription; L: Replication, recombination and repair; D: Cell cycle control, cell division, chromosome partitioning; V: Defense mechanisms; T: Signal transduction mechanisms; M: Cell wall/membrane/envelope biogenesis; N: Cell motility; U: Intracellular trafficking, secretion, and vesicular transport; O: Posttranslational modification, protein turnover, chaperones; C: Energy production and conversion; G: Carbohydrate transport and metabolism; E: Amino acid transport and metabolism; F: Nucleotide transport and metabolism; H: Coenzyme transport and metabolism; I: Lipid transport and metabolism; P: Inorganic ion transport and metabolism; Q: Secondary metabolites biosynthesis, transport and catabolism; X: Mobilome: prophages, transposons; R: General function prediction only; S: Function unknown).

In this study, the expression levels of all genes in the above-mentioned genomes of *Lysinibacillus* were assessed utilizing CAI (Codon Adaption Index) as a numerical estimator. The CAI developed by Sharp et al. [133] measured synonymous codon usage bias for between nucleic acid sequences and confirmed highly expressed reference gene sets, which has been widely applied in many aspects, including estimation of gene expressivity, prediction of highly expressed genes, predicting successful expression likelihood of heterologous gene [134,135,136,137,138,139], and depicting lifestyles of genomes [140]. The results showed that the top four highly expressed COG classes in *L. pakistanensis* LZH-9 based on average CAI values were COG [J] [F] [O] [C] associated with most essential biological processes including nucleotide metabolism, translation, and energy production, followed by COG [M] (cell wall/membrane/envelope biogenesis), [E] (amino acid transport and metabolism), [Q] (secondary metabolites biosynthesis), and [G] (carbohydrate transport and metabolism) reflecting high metabolic rate to digest potential carbon or nitrogen sources in potential contaminants, which closely linked with efficient COD removal ability of LZH-9, whereas genes that were related to COG [X] (mobilome: prophages, transposons) were predicted to be most inactively expressed (Figure 7a). The top four highly expressed COG classes in other strain were mostly consistent with *L. pakistanensis* LZH-9, whereas they differed in the expression levels of other COG classes. In the other two strains of *L. pakistanensis*, genes related to COG [L] (Replication, recombination and repair) in strain JCM 18,776 and genes that were related to COG [V] (Defense mechanisms) in strain UBA7518 were predicted to be most inactively expressed (Figure 7b,c; Figure S41). Principal coordinates analyses (PCoA) of average CAI values based on COG classes were further conducted in order to visualize the similarity or dissimilarity of expression pattern among different strains of the *Lysinibacillus*. Result showed genomes analyzed in this study were clustered into three groups by PCo2 (accounting for 73.4%) based on COG classes (Figure S42), in which strain *L. pakistanensis* LZH-9 were clustered with *L. contaminans* DSM 25560, *L. mangiferihumi* M-GX18 and *L. pakistanensis* JCM 18,776 indicating similar gene expression pattern among these strains, whereas strains *L. parviboronicapiens* VT1065, *L. sphaericus* OT4b.31, and *L. pakistanensis* UBA7518 formed another clusters, and both of the clusters were clearly separated with strain *L. xylanilyticus* t26. It is possible that divergent niches and environmental stresses these strains faced drive differentiation of general gene expression patterns; however, a larger scale examination is needed to support this point of view.

## 4. Conclusions

In this study, we isolated and identified four halotolerant strains from wastewater treatment plant, and found that strain LZH-9 could grow in the presence of up to 14% (*w*/*v*) NaCl, and remove 81.9% COD at 96 h after optimization. Whole genome sequencing of strain LZH-9 and comparative genomic analysis of eight strains of the *Lysinibacillus* revealed metabolic versatility of different genomes of *Lysinibacillus*, and we also found a multitude of genes that were involved in xenobiotics biodegradation, resistance to toxic compounds and salinity in all tested genomes of *Lysinibacillus*, pointing to promising application of *Lysinibacillus* in bioremediation. Genome-scale positive selection analyses showed that those genes under positive selection in *Lysinibacillus* spp. tended to be multifunctional. Additionally, genes that were related to COG [M] [E] [Q] [G] were relatively highly expressed in *L. pakistanensis* LZH-9 in addition to those related to basic biological functions, reflecting high metabolic rate of *L. pakistanensis* to digest potential carbon or nitrogen sources in potential contaminants, which closely linked with efficient COD removal ability of strain LZH-9. In all, the high COD removal efficiency and halotolerance as well as genomic evidences suggested that *L. pakistanensis* LZH-9 possessed great potential to be applied in the bio-treatment of hypersaline industrial wastewater.

## Figures and Tables

**Table 1:** Based on 16S rRNA and chemical oxygen demand (COD) removal efficiency of four halotolerant bacteria.

Strain	Origin	Phylum	Closest Type Strain	Similarity (%)	Salt Tolerance (%)	COD Removal Percentage (%)
LZH-9	Activated sludge	Firmicutes	*Lysinibacillus pakistanensis* JCM 18776^T^	100.00	14	69.8
LZH-13	Activated sludge	Firmicutes	*Pseudomonas hunanensis* LV^T^	98.87	12	57.35
LZH-22	Activated sludge	Firmicutes	*Staphylococcus sciuri* DSM 20345^T^	99.93	20	54.6
LZH-24	Activated sludge	Firmicutes	*Lysinibacillus pakistanensis* JCM 18776^T^	100.00	12	65.43

**Table 2:** For features of the eight *Lysinibacillus* genomes involved in this study.

Organism/Name	Strain	Genbank or IMG Acc. no.	Level	Completeness (%)	Size (Mb)	Coding Density (%)	GC (%)	Gene	Protein	Source	Reference
*Lysinibacillus pakistanensis*	JCM 18776	GCA_001312325.1 /2728369720	Draft	99.1	5.01	77.7	36.3	8328	8216	rhizospheric soil of legume	[60]
*Lysinibacillus contaminans*	DSM 25560	GCA_001278945.1 /2648501842	Draft	98.1	4.1	82.2	37.3	3879	3666	Surface water	[10]
*Lysinibacillus xylanilyticus*	t26	GCA_002803495.1	Draft	99.4	5.69	79.9	36.8	6045	5584	/	/
*Lysinibacillus* sp.	UBA7518	GCA_002478295.1	Draft	88.6	4.06	71.5	37.8	3969	3915	/	/
*Lysinibacillus sphaericus*	OT4b.31	GCA_000392615.1 /2524614537	Draft	99.2	4.86	81.5	37.5	4687	4441	Beetle larvae	[10]
*Lysinibacillus mangiferihumi*	M-GX18	GCA_003049665.1 /2791355086	Draft	99.4	5.31	82.6	36.7	5590	5187	/	/
*Lysinibacillus pakistanensis*	LZH-9	GCA_009649975.1 /2823662158	Complete	100	4.99	83.7	36.6	5263	4990	Activated sludge	This study.
*Lysinibacillus parviboronicapiens*	VT1065	GCA_003049605.1 /2802429645	Draft	98.3	4.74	82	37.5	4676	4351	/	/

## Supplementary Materials

The following are available online at https://www.mdpi.com/2076-2607/8/5/716/s1, Table S1: GO enrichment analyses (*p*-value < 0.05) of gene families in six representative strains of *Lysinibacillus*. Table S2: Numbers of cytochrome P450 and peptidases in genomes of *Lysinibacillus* spp., Table S3: Statistics of Mobile genetic elements (MGEs) present in the genomes of *Lysinibacillus* spp. Table S4: Genes under positive selection of *Lysinibacillus* detected by posigene pipeline, Figure S1: Heat map of whole genome BLASTN-based average nucleotide identity (ANI) value of nine strains of genus *Lysinibacillus*, Figure S2: BlastN-based whole genome comparisons of eight representative strains of genus *Lysinibacillus* using BRIG and *L. pakistanensis* LZH-9 was used as reference, Figure S3: BlastN-based whole genome comparison of strains *L. pakistanensis* JCM 18776, *L. contaminans* DSM 25560, *L. xylanilyticus* t26, *Lysinibacillus* sp. UBA7518, *L. sphaericus* OT4b.31, *L. mangiferihumi* M-GX18 and *L. parviboronicapiens* VT1065, *L. pakistanensis* LZH-9, Figure S4: Bar chart showing proportions of COG classes of different part of 3 strains of *L. pakistanensis* (*L. pakistanensis* JCM 18776, *Lysinibacillus* sp. UBA7518, *L. pakistanensis* LZH-9) pan-genome (i.e., core, accessory, unique), Figure S5: Mathematical modeling of the pan-genome and core genome of 6 strains of *Lysinibacillus* type strains. *L. contaminans* DSM 25560, *L. xylanilyticus* t26, *L. sphaericus* OT4b.31, *L. mangiferihumi* M-GX18 and *L. parviboronicapiens* VT1065, *L. pakistanensis* LZH-9, Figure S6: Bar chart showing proportions of COG classes of different part of 6 strains of *Lysinibacillus* type strains pan-genome (i.e., core, accessory, unique), Figure S7: Maximum likelihood phylogenetic tree of mannose-6-phosphate isomerase protein sequences derived from *Lysinibacillus* spp. strains and other representative species, Figure S8: Maximum likelihood phylogenetic tree of gluconate 2-dehydrogenase protein sequences derived from *Lysinibacillus* spp. strains and other representative species, Figure S9: Maximum likelihood phylogenetic tree of ribokinase protein sequences derived from *Lysinibacillus* spp. strains and other representative species, Figure S10: Maximum likelihood phylogenetic tree of glycerophosphodiester phosphodiesterase protein sequences derived from *Lysinibacillus* spp. strains and other representative species, Figure S11: Maximum likelihood phylogenetic tree of vancomycin B-type resistance proteins VanW sequences derived from *Lysinibacillus* spp. strains and other representative species, Figure S12: Maximum likelihood phylogenetic tree of vancomycin response regulator VanR sequences derived from *Lysinibacillus* spp. strains and other representative species, Figure S13: Maximum likelihood phylogenetic tree of vancomycin sensor histidine kinase VanS sequences derived from *Lysinibacillus* spp. strains and other representative species, Figure S14: Maximum likelihood phylogenetic tree of fosmidomycin resistance protein FosB sequences derived from *Lysinibacillus* spp. strains and other representative species, Figure S15: Maximum likelihood phylogenetic tree of N-acetyltransferases SatA sequences derived from *Lysinibacillus* spp. strains and other representative species, Figure S16: Maximum likelihood phylogenetic tree of beta-lactamases sequences derived from *Lysinibacillus* spp. strains and other representative species, Figure S17: Maximum likelihood phylogenetic tree of Colicin E2 tolerance protein CbrC sequences derived from *Lysinibacillus* spp. strains and other representative species, Figure S18: Maximum likelihood phylogenetic tree of MATE (Multidrug and Toxic Compound Extrusion) family efflux protein sequences derived from *Lysinibacillus* spp. strains and other representative species, Figure S19: Maximum likelihood phylogenetic tree of arsenite efflux pump Acr3 sequences derived from *Lysinibacillus* spp. strains and other representative species, Figure S20: Maximum likelihood phylogenetic tree of arsenite efflux pump ArsB sequences derived from *Lysinibacillus* spp. strains and other representative species, Figure S21: Maximum likelihood phylogenetic tree of ATP-dependent efflux systems component CadA sequences derived from *Lysinibacillus* spp. strains and other representative species, Figure S22: Maximum likelihood phylogenetic tree of ATP-dependent efflux systems component CadC sequences derived from *Lysinibacillus* spp. strains and other representative species, Figure S23: Maximum likelihood phylogenetic tree of cation diffusion facilitator (CDF) protein CzcD sequences derived from *Lysinibacillus* spp. strains and other representative species, Figure S24: Maximum likelihood phylogenetic tree of S-ribosylhomocysteinase (LuxS) sequences derived from *Lysinibacillus* spp. strains and other representative species, Figure S25: Maximum likelihood phylogenetic tree of potassium transporting ATPase subunit A (KdpA) sequences derived from *Lysinibacillus* spp. strains and other representative species, Figure S26: Maximum likelihood phylogenetic tree of potassium transporting ATPase subunit B (KdpB) sequences derived from *Lysinibacillus* spp. strains and other representative species, Figure S27: Maximum likelihood phylogenetic tree of potassium transporting ATPase subunit C (KdpC) sequences derived from *Lysinibacillus* spp. strains and other representative species, Figure S28: Maximum likelihood phylogenetic tree of osmosensitive K^+^ channel histidine kinases KdpD sequences derived from *Lysinibacillus* spp. strains and other representative species, Figure S29: Maximum likelihood phylogenetic tree of cytosolic octameric regulatory protein (KtrA) sequences derived from *Lysinibacillus* spp. and other representative species, Figure S30: Maximum likelihood phylogenetic tree of choline dehydrogenase (BetA) sequences derived from *Lysinibacillus* spp. strains and other representative species, Figure S31: Maximum likelihood phylogenetic tree of ATP-binding cassette transporter component OpuAA sequences derived from *Lysinibacillus* spp. strains and other representative species, Figure S32: Maximum likelihood phylogenetic tree of ATP-binding cassette transporter component OpuAB sequences derived from *Lysinibacillus* spp. strains and other representative species, Figure S33: Maximum likelihood phylogenetic tree of ATP-binding cassette transporter component OpuAC sequences derived from *Lysinibacillus* spp. strains and other representative species, Figure S34: Maximum likelihood phylogenetic tree of Acetyl-CoA C-acetyltransferase (AtoB) sequences derived from *Lysinibacillus* spp. strains and other representative species, Figure S35: Maximum likelihood phylogenetic tree of 3-hydroxybutyryl-CoA dehydrogenase (FadB) sequences derived from *Lysinibacillus* spp. strains and other representative species, Figure S36: Maximum likelihood phylogenetic tree of 3-hydroxyacyl-CoA dehydrogenase/enoyl-CoA hydratase (FadJ) sequences derived from *Lysinibacillus* spp. strains and other representative species, Figure S37: Maximum likelihood phylogenetic tree of 4-nitrophenyl phosphatase sequences derived from *Lysinibacillus* spp. strains and other representative species, Figure S38: Maximum likelihood phylogenetic tree of nitrilotriacetate monooxygenase component B sequences derived from *Lysinibacillus* spp. strains and other representative species, Figure S39: Synteny analysis of a xenobiotics biodegradation and metabolism related gene cluster *dmpR-hpaB-dmpB-pnpA-mhpD-dmpFGH-xylH-hpaE* derived from *L. pakistanensis* LZH-9, and other representative species in *Lysinibacillus* and GC contents comparison against the genome of *L. pakistanensis* LZH-9, Figure S40: Scatterplots of the relationship between: (a) the numbers of transposons sequences and total length of transposons, (b) the numbers of genomic island sequences and total length of genomic island, (c) the numbers of phage sequences and total length of phage region, (d) the numbers of CRISPR-Cas sequences and total length of CRISPR-Cas sequences detected in genomes in Table 2, Figure S41: Ranges of CAI values of different genes in the genome of five strains belonged *Lysinibacillus* based on COG classification. (a) *L. sphaericus* OT4b.31; (b) *L. contaminans* DSM 25560; (c) *L. parviboronicapiens* VT1065; (d) *L. mangiferihumi* M-GX18; (e) *L. xylanilyticus* t26, Figure S42: Principal coordinates analyses (PCoA) of average CAI values based on COG classes were conducted to visualize the similarity or dissimilarity of expression pattern among different strains of the *Lysinibacillus* (1: *L. sphaericus* OT4b.31, 2: *L. contaminans* DSM 25560, 3: *L. parviboronicapiens* VT1065, 4: *L. mangiferihumi* M-GX18, 5: *L. xylanilyticus* t26, 6: *L. pakistanensis* JCM 18776, 7: *Lysinibacillus* sp. UBA7518, 8: *L. pakistanensis* LZH-9). Result showed strains in this study were clustered into three groups (ABC) by PCo2 (accounting for 73.4%) based on COG classes.
