# Supplementary material for: Whole Genome Sequencing and Comparative Genomic Analyses of Lysinibacillus pakistanensis LZH-9, a Halotolerant Strain with Excellent COD Removal Capability
Source: Microorganisms. 2020 May 12;8(5):716. doi: 10.3390/microorganisms8050716 (PMC7284689; doi:10.3390/microorganisms8050716)
Supplement: Supplementary file 1 [file microorganisms-08-00716-s001.zip › Supplementary Materials/Supplementary Materials 1.docx]

*Supplementary Material*

**Table S2** Numbers of cytochrome P450 and peptidases in genomes of *Lysinibacillus* spp.

| Strain | Numbers of Cytochrome P450 | Numbers of Peptidases |
| --- | --- | --- |
| *L. contaminans* DSM 25560 | 32 | 108 |
| *L. mangiferihumi* M-GX18 | 35 | 133 |
| *L. pakistanensis* JCM 18776 | 66 | 287 |
| *Lysinibacillus* sp. UBA7518 | 32 | 122 |
| *L. pakistanensis* LZH-9 | 43 | 159 |
| *L. parviboronicapiens* VT1065 | 40 | 138 |
| *L. sphaericus* OT4b.31 | 35 | 140 |
| *L. xylanilyticus* t26 | 44 | 138 |

**Table S4** **Genes under positive selection of *Lysinibacillus* detected by posigene pipeline.**

|  |  |  |  |  |  |
| --- | --- | --- | --- | --- | --- |
| Gene | FDR | P-Value | COG class | Gene name | Product |
| Lp_1054 | 0.0004 | 7.35E-06 | N | *-* | Uncharacterized conserved protein |
| Lp_718 | 0.0032 | 1.18E-04 | I | *fabB* | β-ketoacyl-ACP synthase |
| Lp_3474 | 0.0174 | 1.10E-03 | E | *trpF* | Phosphoribosylanthranilate isomerase |
| Lp_3540 | 0.0174 | 1.26E-03 | - | *-* | - |
| Lp_2135 | 0.0322 | 2.93E-03 | - | *-* | - |
| Lp_4098 | 0.0354 | 3.87E-03 | O | *surA* | Parvulin-like peptidyl-prolyl isomerase |
| Lp_411 | 0.0386 | 4.92E-03 | R | *thiJ* | Putative intracellular protease/amidase |
|  |  |  |  |  |  |

**
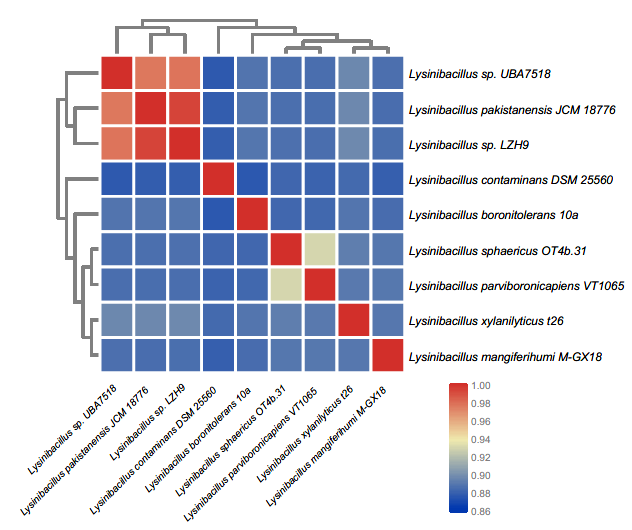
**

**Figure S1.** Heat map of whole genome BLASTN-based average nucleotide identity (ANI) value of nine strains of genus *Lysinibacillus.*

**
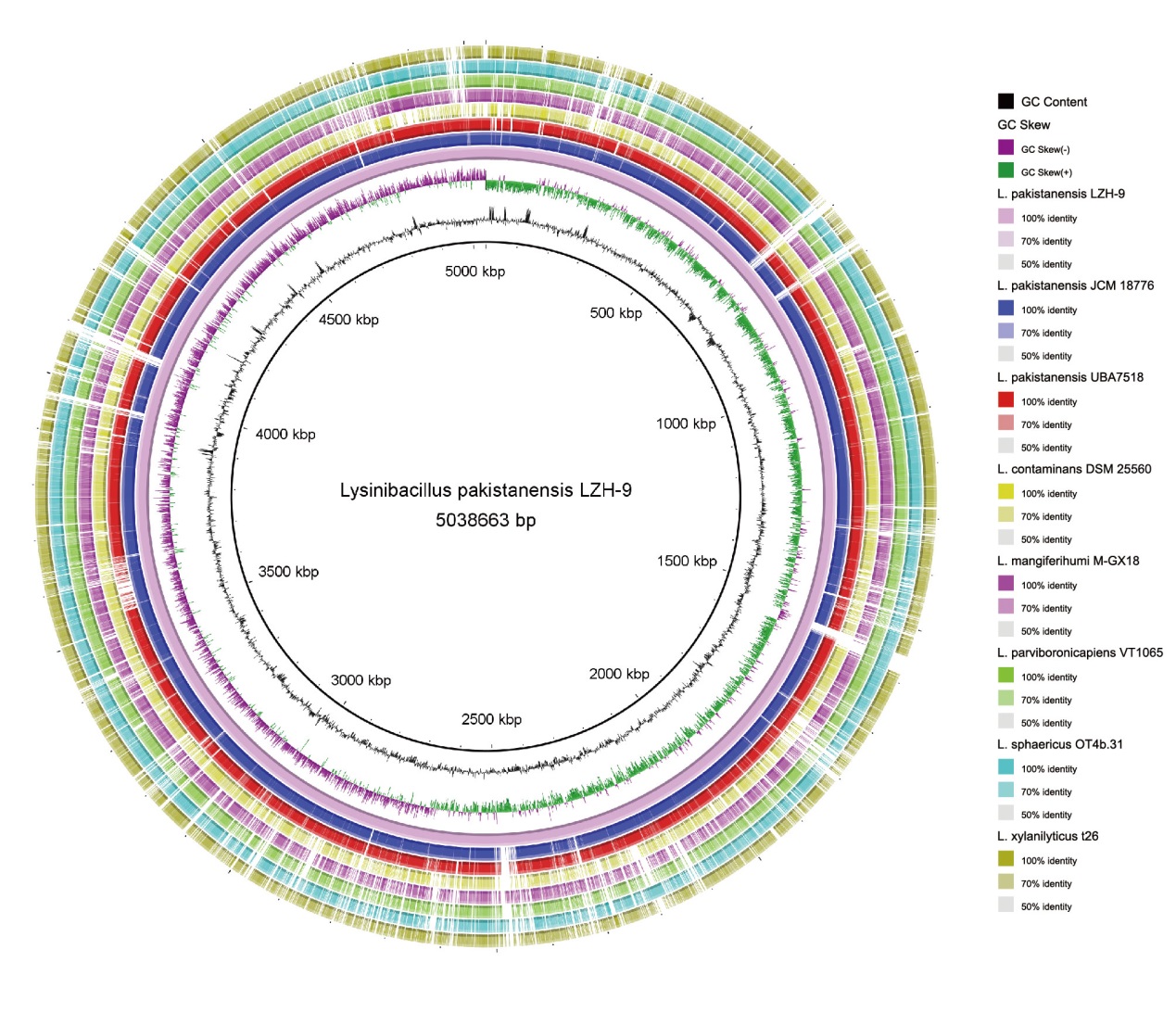
**

**Figure S2.** BlastN-based whole genome comparisons of eight representative strains of genus *Lysinibacillus* using BRIG and *L. pakistanensis* LZH-9 was used as reference.


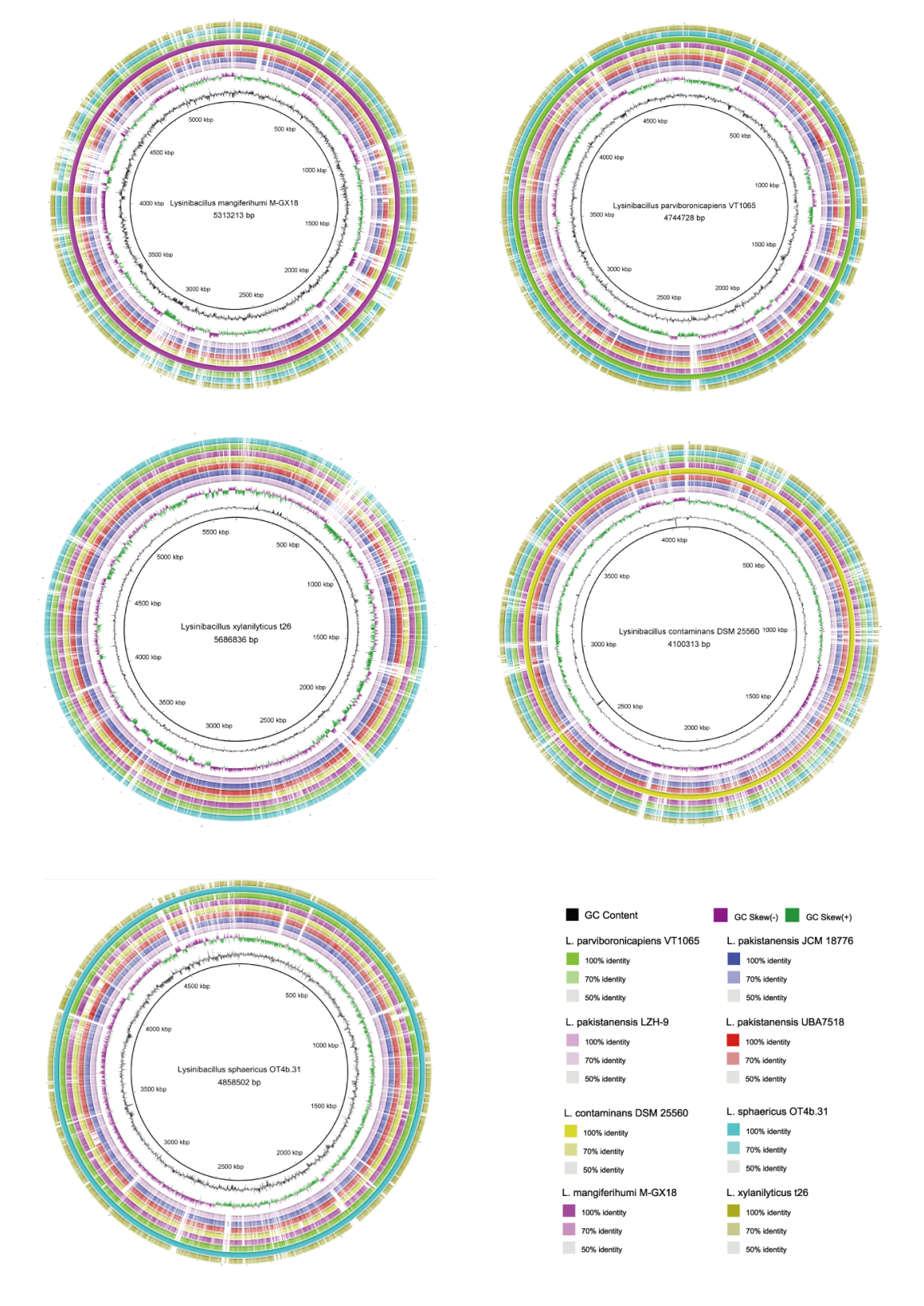


**Figure S3.** BlastN-based whole genome comparison of strains *L. pakistanensis* JCM 18776, *L. contaminans* DSM 25560, *L. xylanilyticus* t26，*Lysinibacillus* sp. UBA7518, *L. sphaericus* OT4b.31, *L. mangiferihumi* M-GX18 and *L. parviboronicapiens* VT1065, *L. pakistanensis* LZH-9.


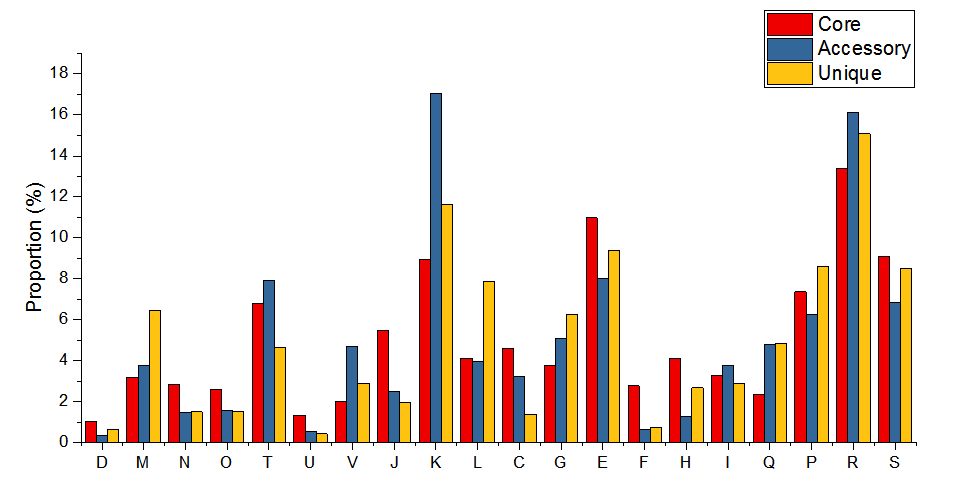


**Figure S4.** Bar chart showing proportions of COG classes of different part of 3 strains of *L. pakistanensis* (*L. pakistanensis* JCM 18776, *Lysinibacillus* sp. UBA7518, *L. pakistanensis* LZH-9) pan-genome (i.e., core, accessory, unique).


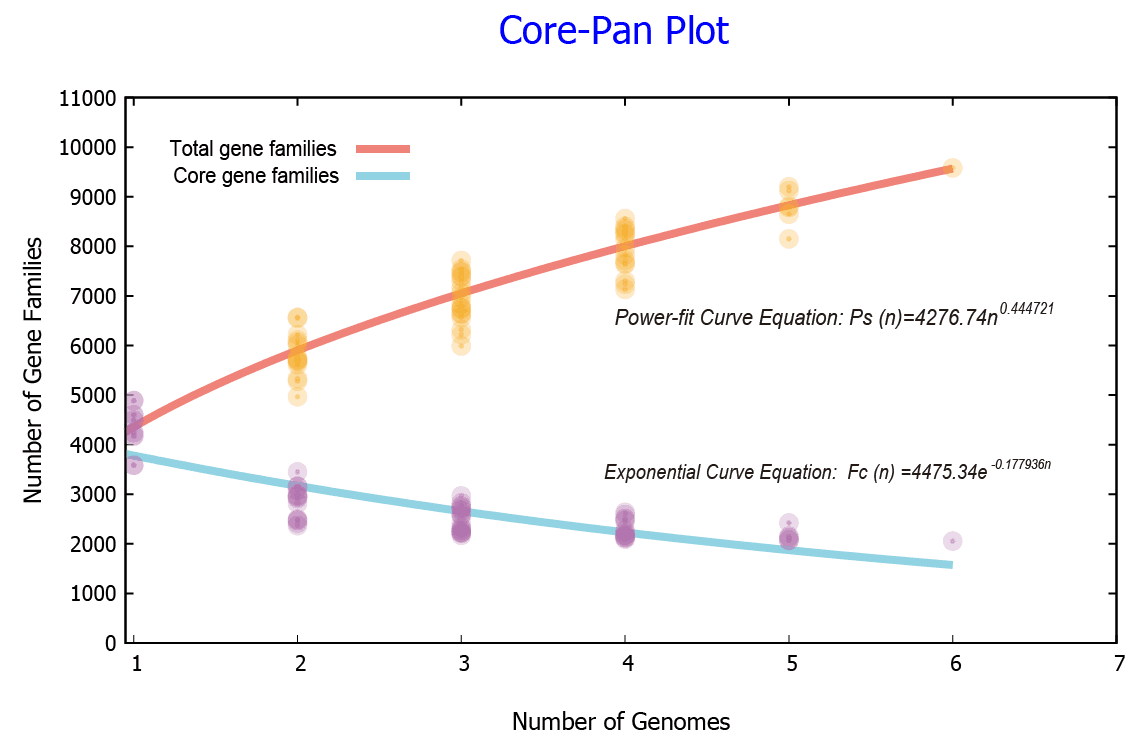


**Figure S5.** Mathematical modeling of the pan-genome and core genome of 6 strains of *Lysinibacillus* representative strains. *L. contaminans* DSM 25560, *L. xylanilyticus* t26, *L. sphaericus* OT4b.31, *L. mangiferihumi* M-GX18 and *L. parviboronicapiens* VT1065, *L. pakistanensis* LZH-9.


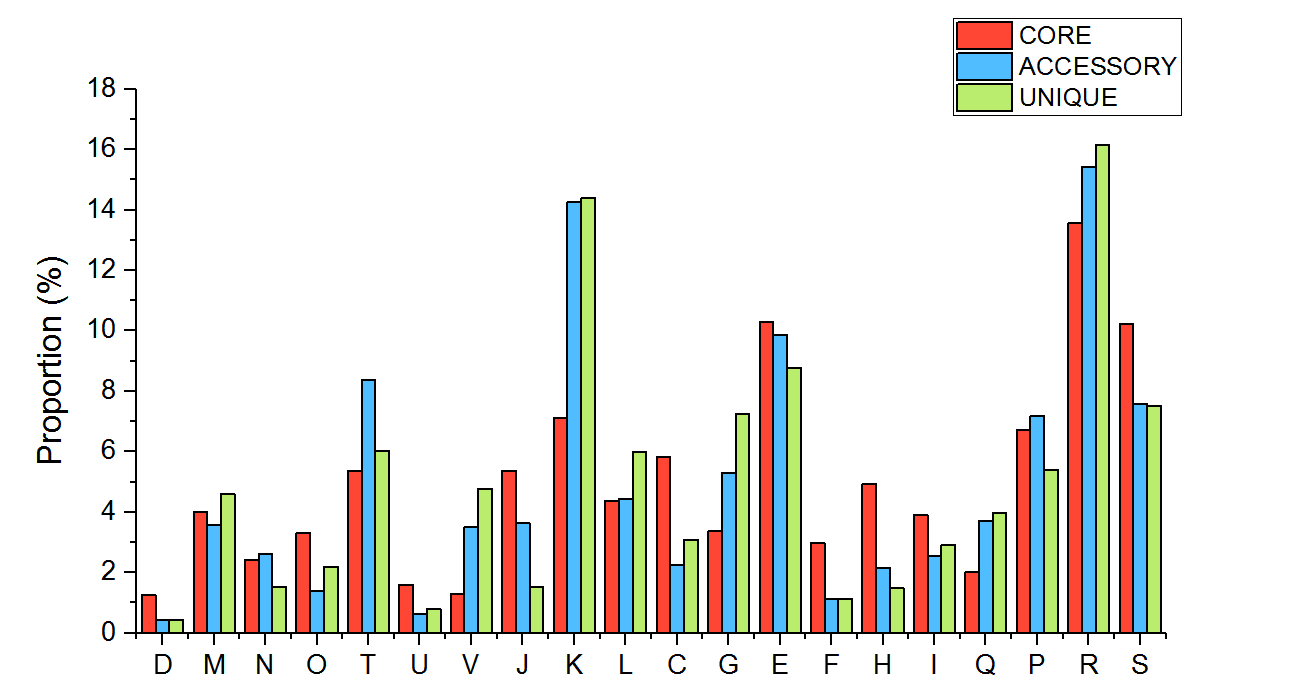


**Figure S6.** Bar chart showing proportions of COG classes of different part of 6 strains of *Lysinibacillus* representative strains pan-genome (i.e., core, accessory, unique).


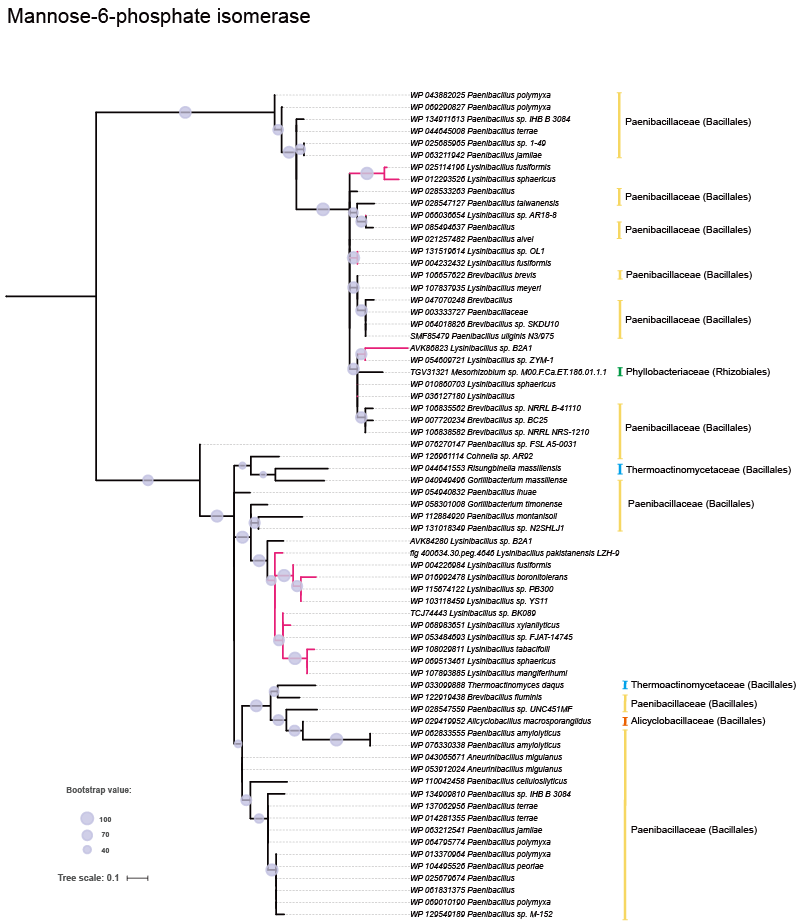
**Figure S7.** Maximum likelihood phylogenetic tree of mannose-6-phosphate isomerase protein sequences derived from *Lysinibacillus* spp. strains and other representative species.


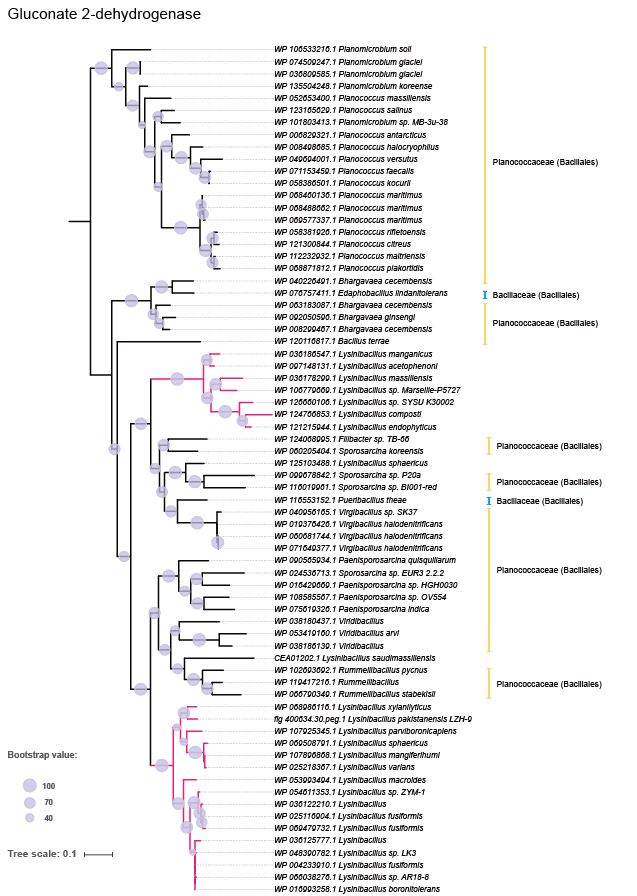


**Figure S8.** Maximum likelihood phylogenetic tree of gluconate 2-dehydrogenase protein sequences derived from *Lysinibacillus* spp. strains and other representative species.


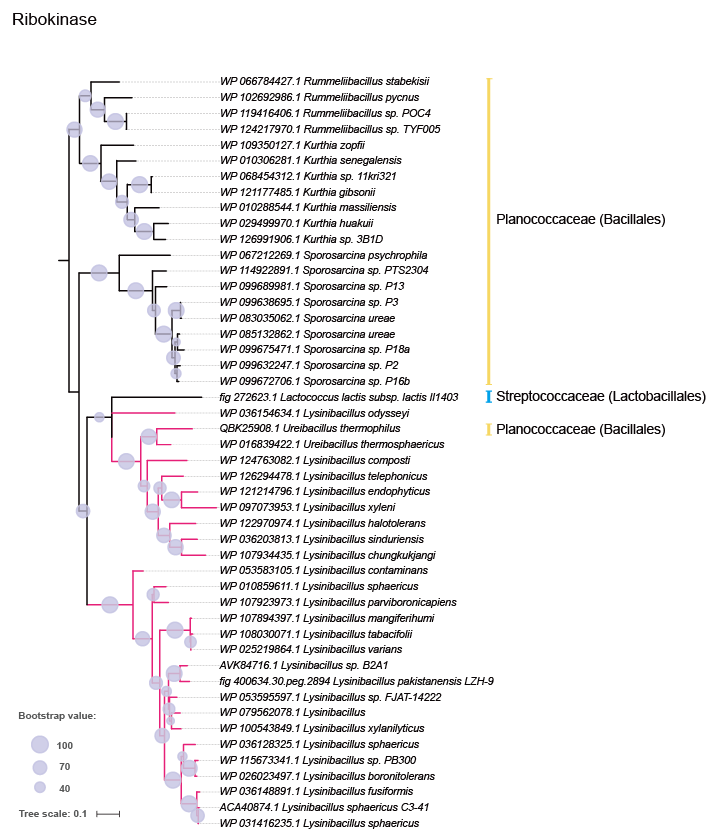


**Figure S9.** Maximum likelihood phylogenetic tree of ribokinase protein sequences derived from *Lysinibacillus* spp. strains and other representative species.


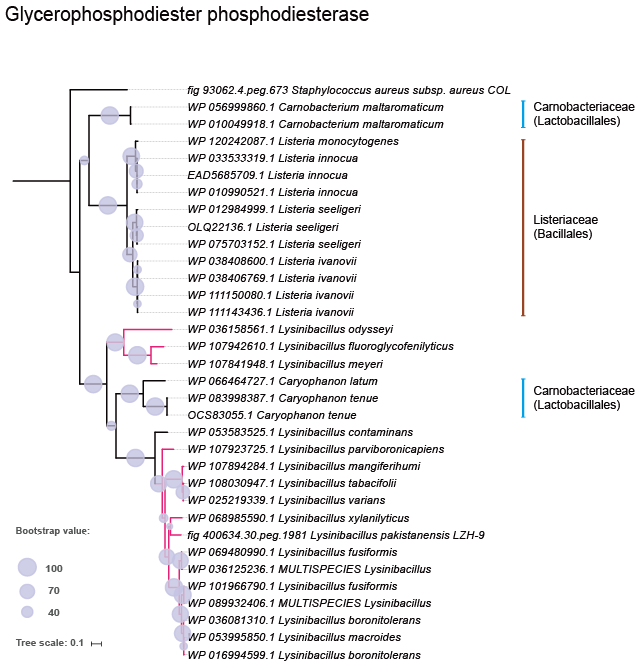


**Figure S10.** Maximum likelihood phylogenetic tree of glycerophosphodiester phosphodiesterase protein sequences derived from *Lysinibacillus* spp. strains and other representative species.


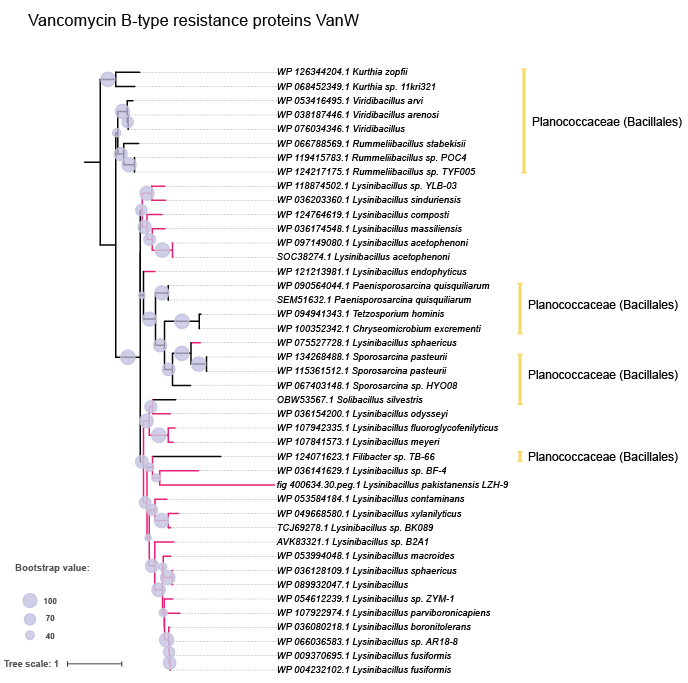
**Figure S11.** Maximum likelihood phylogenetic tree of vancomycin B-type resistance proteins VanW sequences derived from *Lysinibacillus* spp. strains and other representative species.


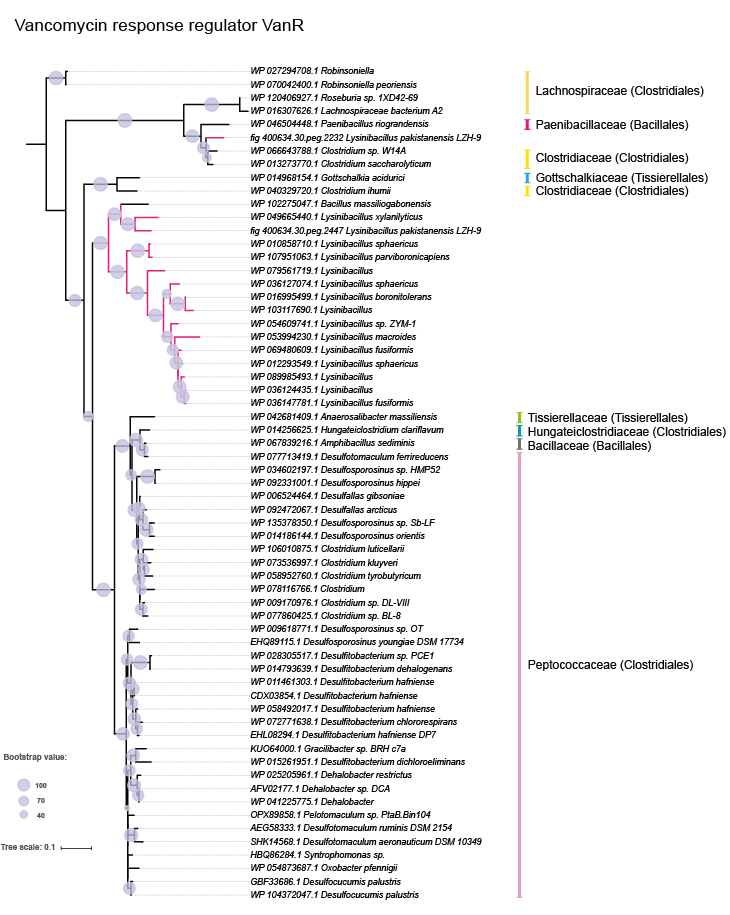


**Figure S12.** Maximum likelihood phylogenetic tree of vancomycin response regulator VanR sequences derived from *Lysinibacillus* spp. strains and other representative species.

**
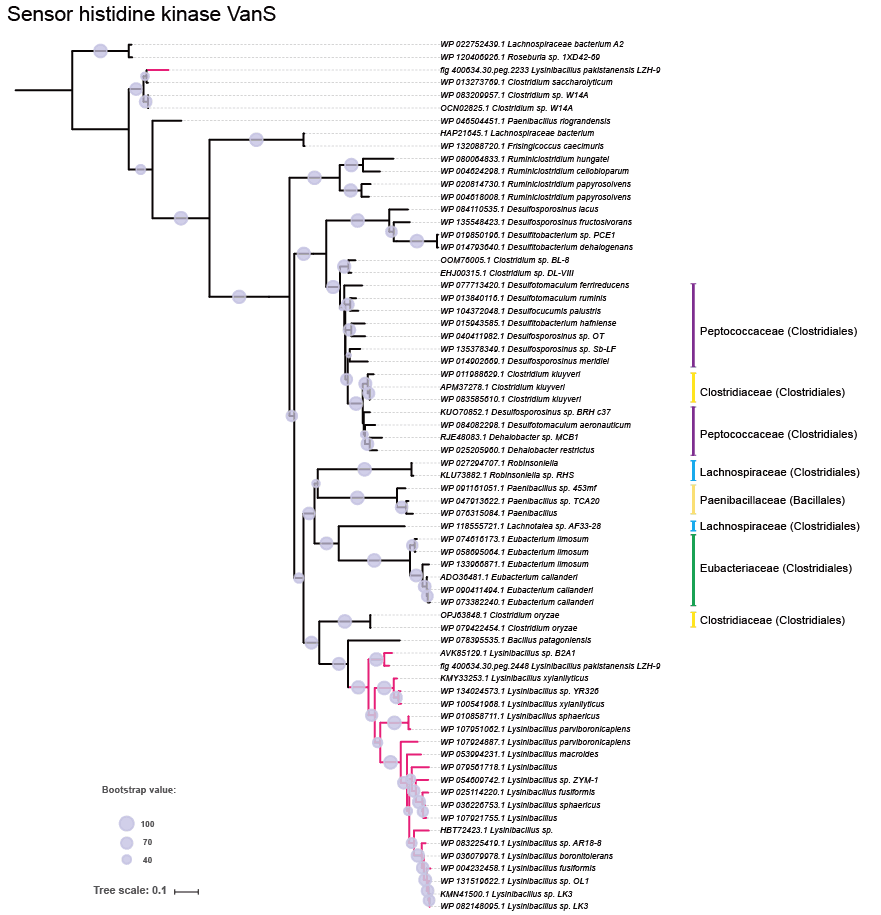
**

**Figure S13.** Maximum likelihood phylogenetic tree of vancomycin sensor histidine kinase VanS sequences derived from *Lysinibacillus* spp. strains and other representative species.


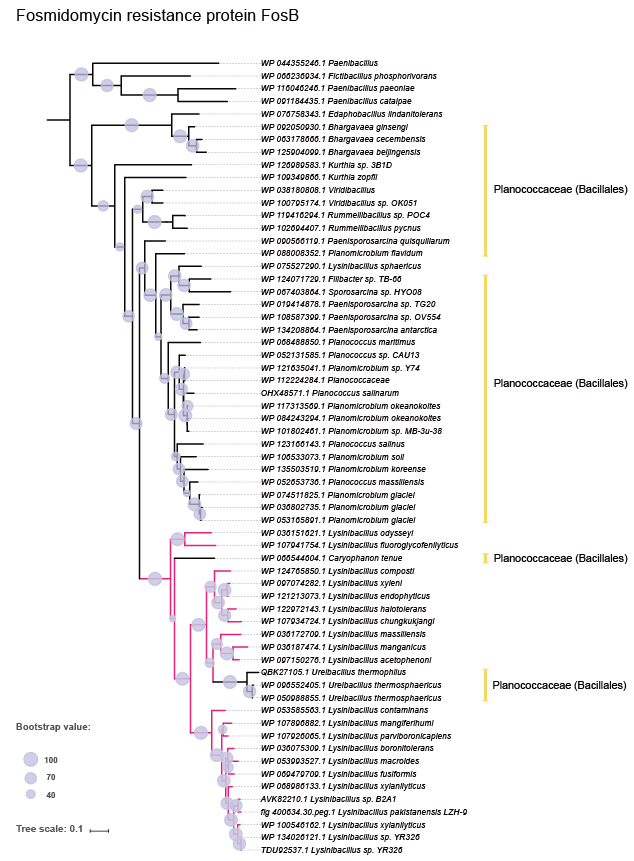
**Figure S14.** Maximum likelihood phylogenetic tree of fosmidomycin resistance protein FosB sequences derived from *Lysinibacillus* spp. strains and other representative species.

**
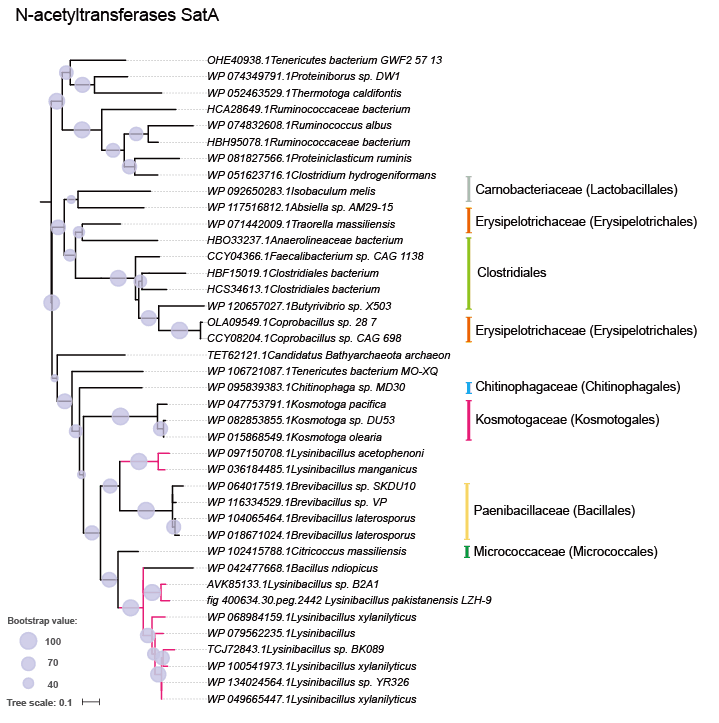
**

**Figure S15.** Maximum likelihood phylogenetic tree of N-acetyltransferases SatA sequences derived from *Lysinibacillus* spp. strains and other representative species.


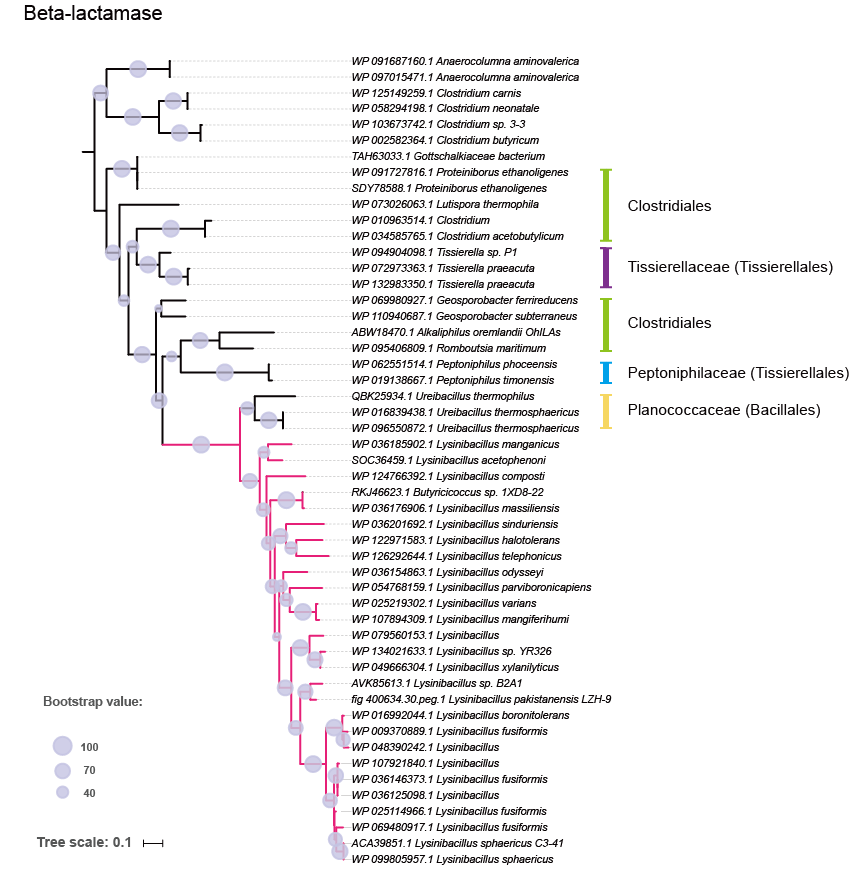


**Figure S16.** Maximum likelihood phylogenetic tree of beta-lactamases sequences derived from *Lysinibacillus* spp. strains and other representative species.


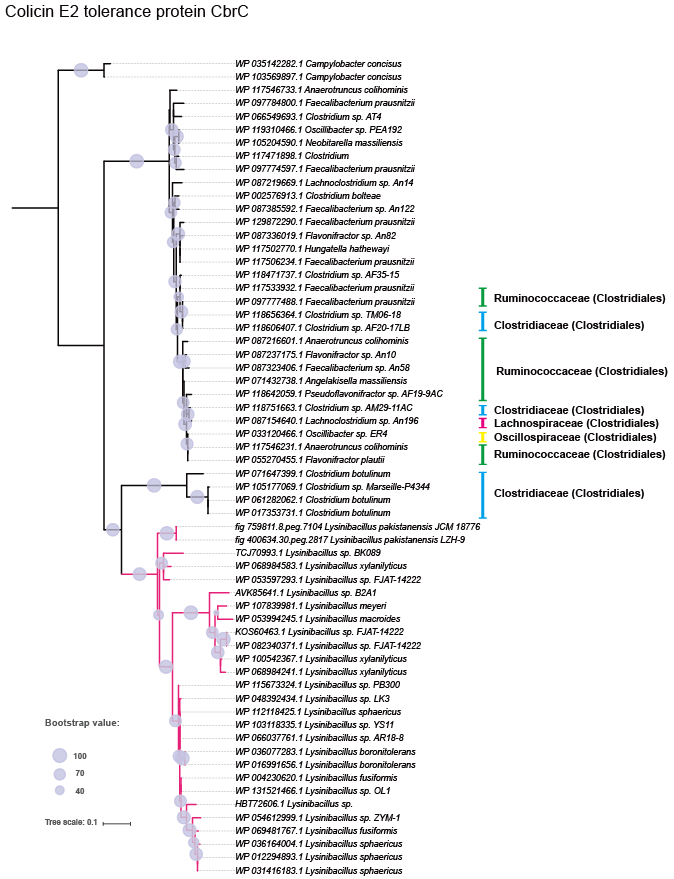


**Figure S17.** Maximum likelihood phylogenetic tree of Colicin E2 tolerance protein CbrC sequences derived from *Lysinibacillus* spp. strains and other representative species.


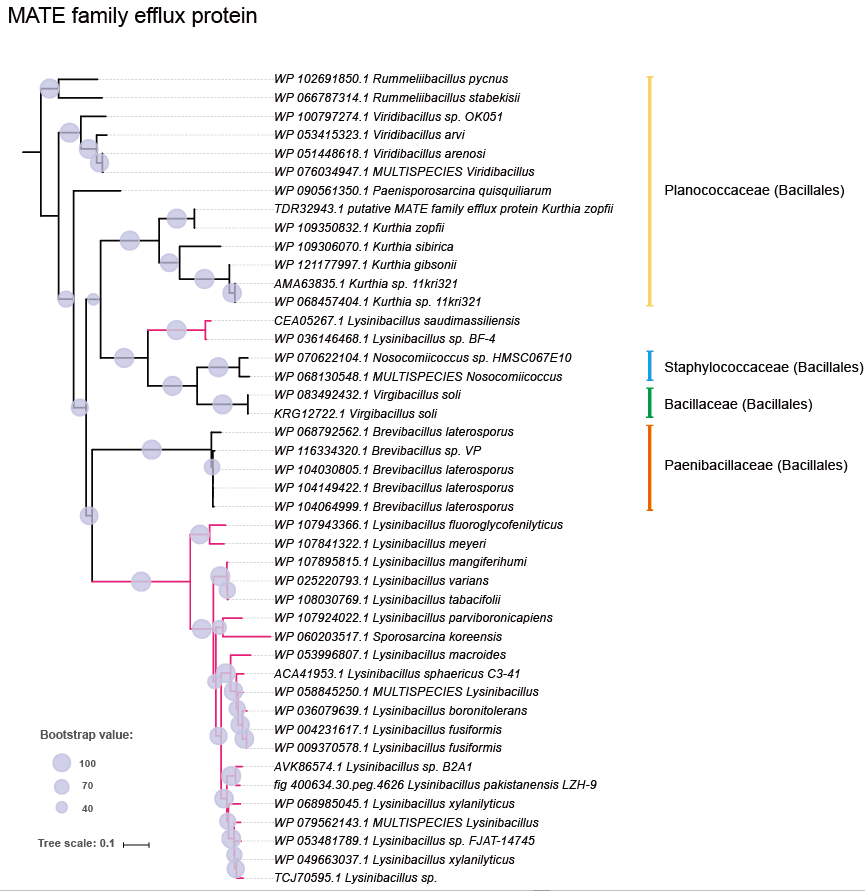


**Figure S18.** Maximum likelihood phylogenetic tree of MATE (Multidrug and Toxic Compound Extrusion) family efflux protein sequences derived from *Lysinibacillus* spp. strains and other representative species.

**Figure S19.** Maximum likelihood phylogenetic tree of arsenite efflux pump Acr3 sequences derived from *Lysinibacillus* spp. strains and other representative species.


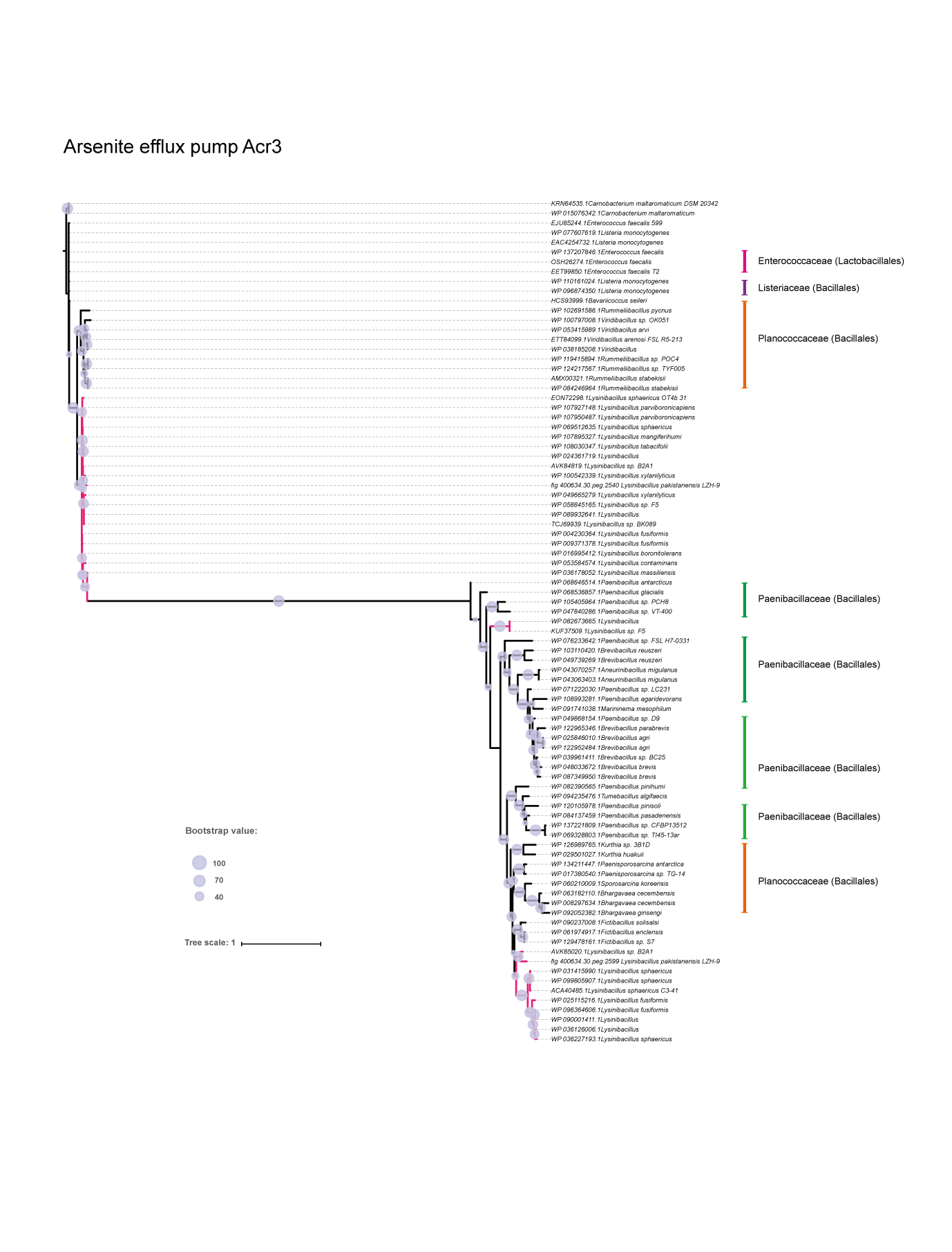


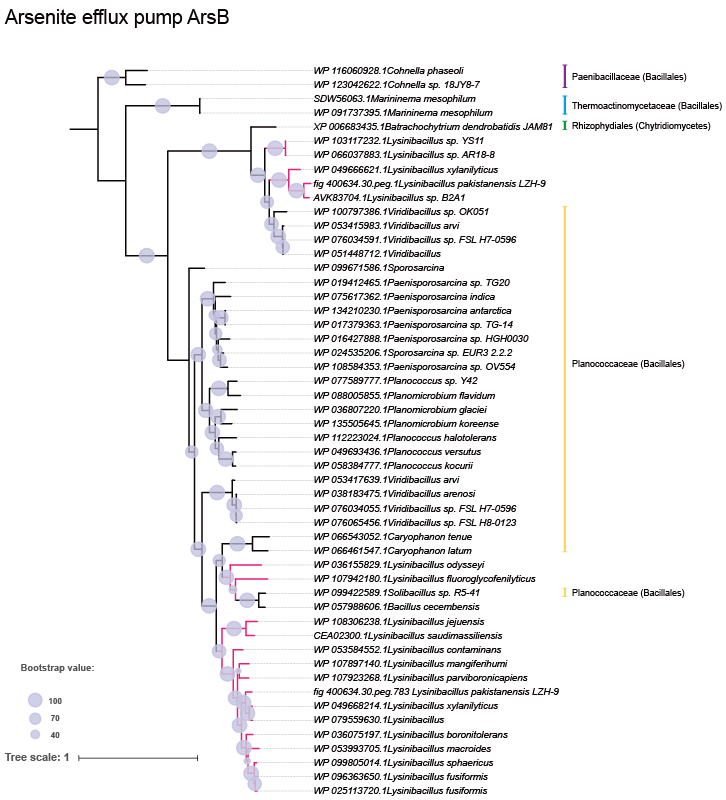


**Figure S20.** Maximum likelihood phylogenetic tree of arsenite efflux pump ArsB sequences derived from *Lysinibacillus* spp. strains and other representative species.


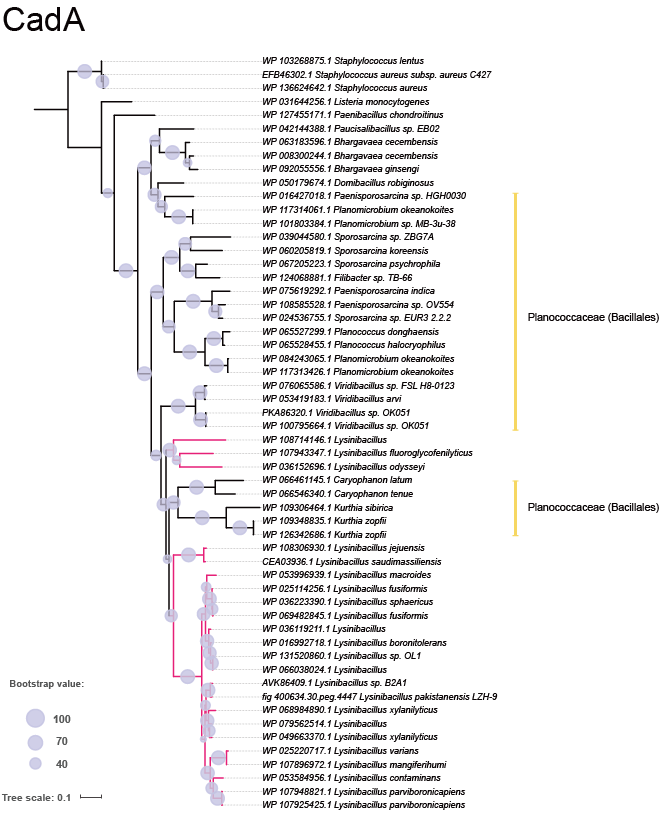


**Figure S21.** Maximum likelihood phylogenetic tree of ATP-dependent efflux systems component CadA sequences derived from *Lysinibacillus* spp. strains and other representative species.


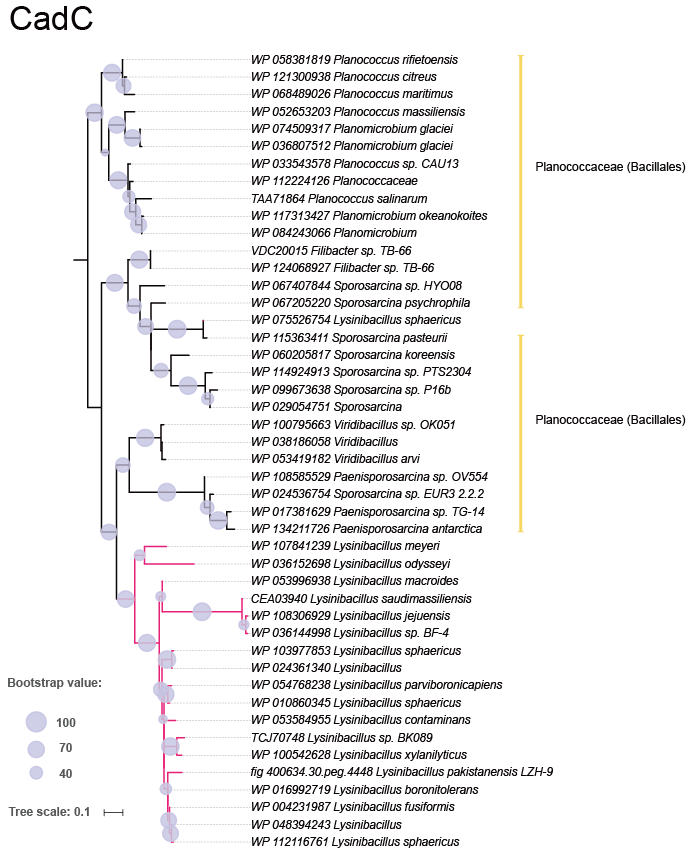


**Figure S22.** Maximum likelihood phylogenetic tree of ATP-dependent efflux systems component CadC sequences derived from *Lysinibacillus* spp. strains and other representative species.


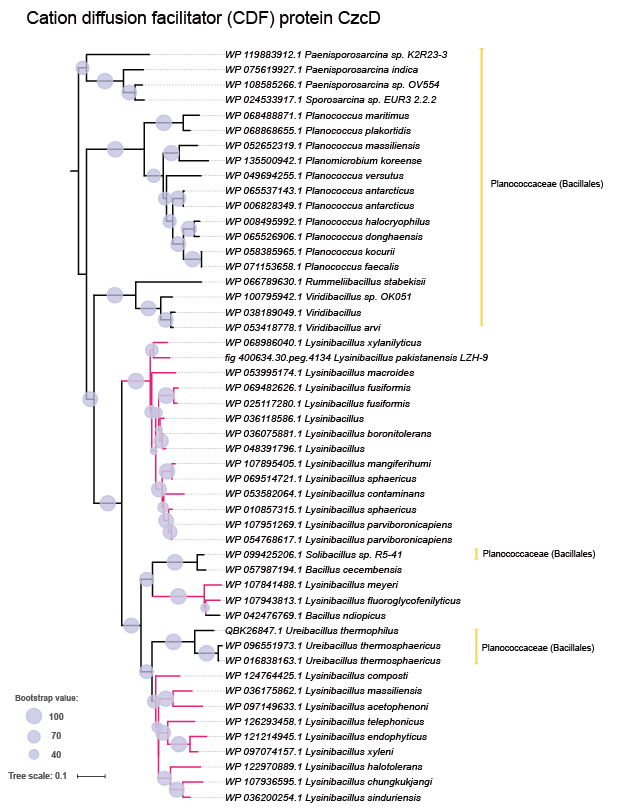


**Figure S23.** Maximum likelihood phylogenetic tree of cation diffusion facilitator (CDF) protein CzcD sequences derived from *Lysinibacillus* spp. strains and other representative species.


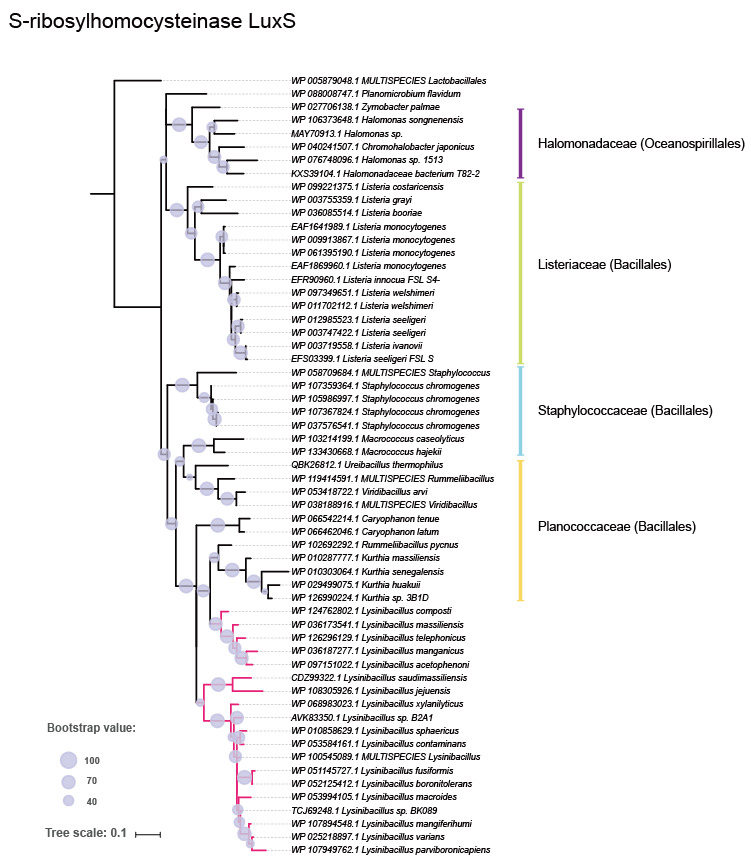


**Figure S24.** Maximum likelihood phylogenetic tree of S-ribosylhomocysteinase (LuxS) sequences derived from *Lysinibacillus* spp. strains and other representative species.


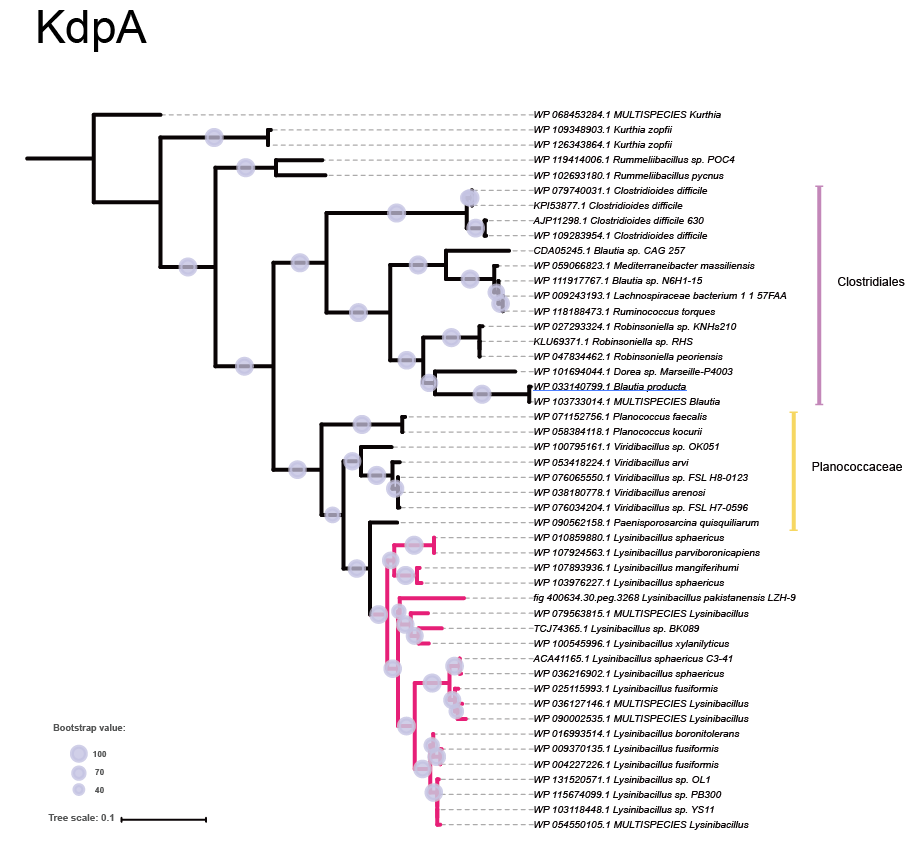


**Figure S25.** Maximum likelihood phylogenetic tree of potassium transporting ATPase subunit A (KdpA) sequences derived from *Lysinibacillus* spp. strains and other representative species.


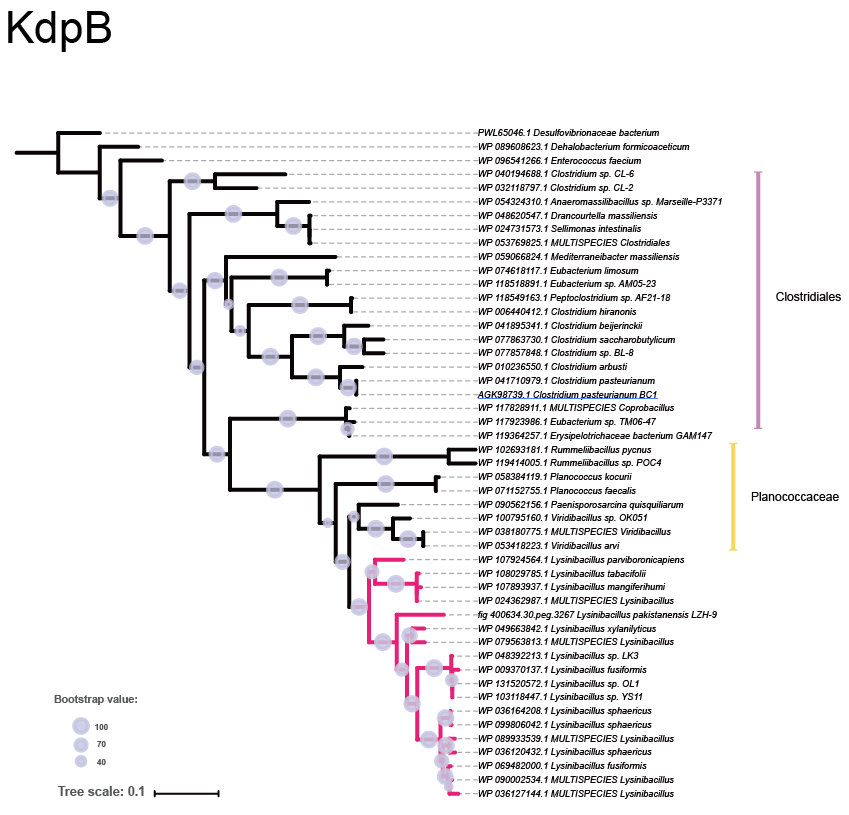


**Figure S26.** Maximum likelihood phylogenetic tree of potassium transporting ATPase subunit B (KdpB) sequences derived from *Lysinibacillus* spp. strains and other representative species.


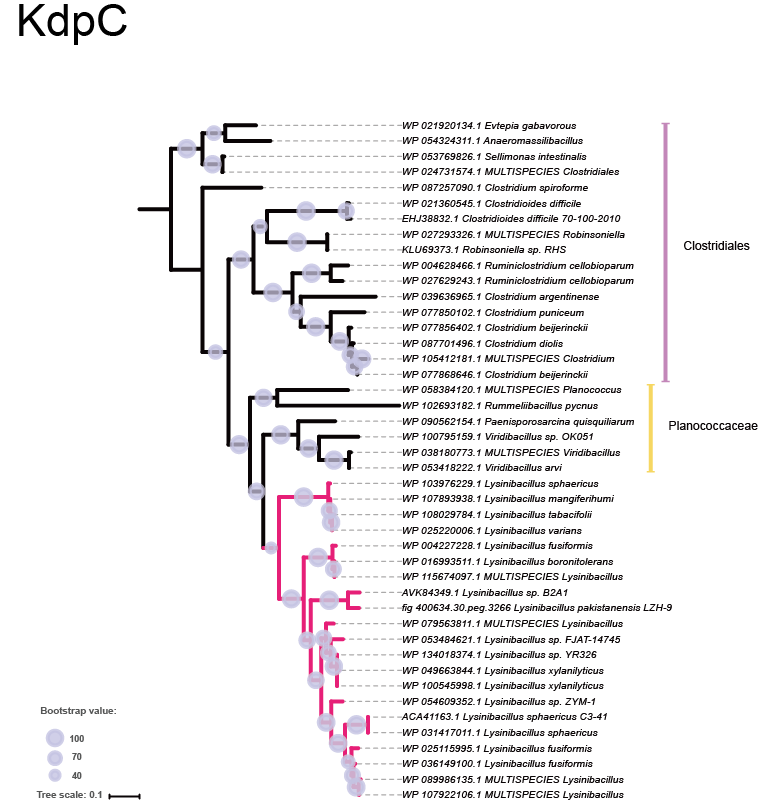


**Figure S27.** Maximum likelihood phylogenetic tree of potassium transporting ATPase subunit C (KdpC) sequences derived from *Lysinibacillus* spp. strains and other representative species.


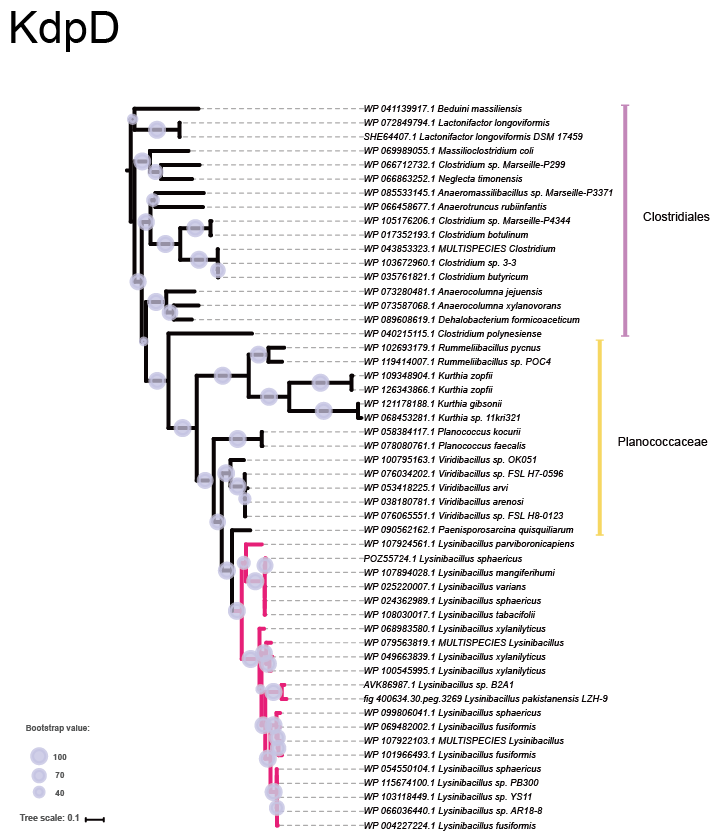


**Figure S28.** Maximum likelihood phylogenetic tree of osmosensitive K^+^ channel histidine kinases KdpD sequences derived from *Lysinibacillus* spp. strains and other representative species.


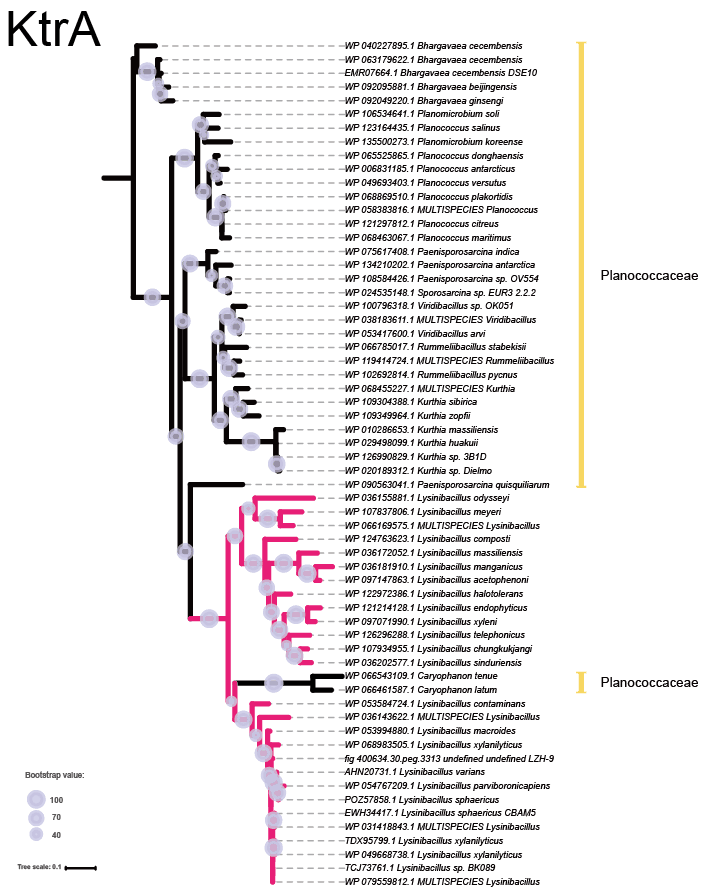


**Figure S29.** Maximum likelihood phylogenetic tree of cytosolic octameric regulatory protein (KtrA) sequences derived from *Lysinibacillus* spp. and other representative species.


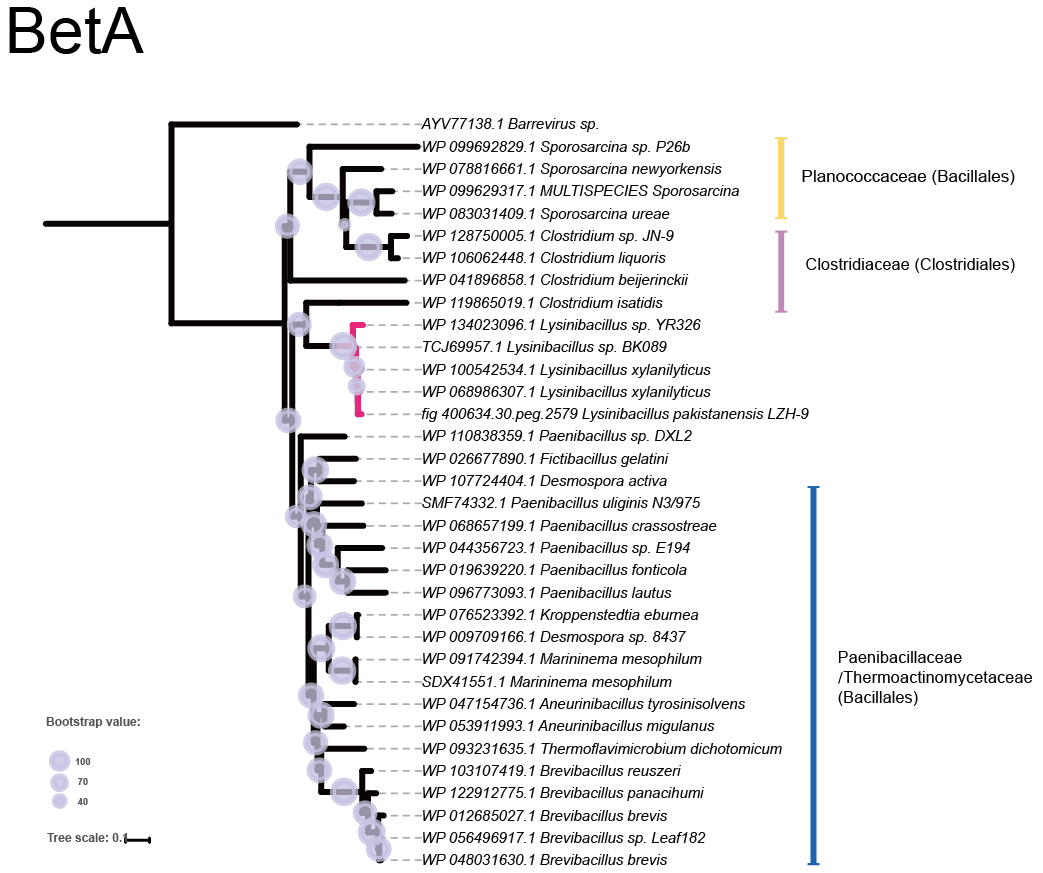


**Figure S30.** Maximum likelihood phylogenetic tree of choline dehydrogenase (BetA) sequences derived from *Lysinibacillus* spp. strains and other representative species.


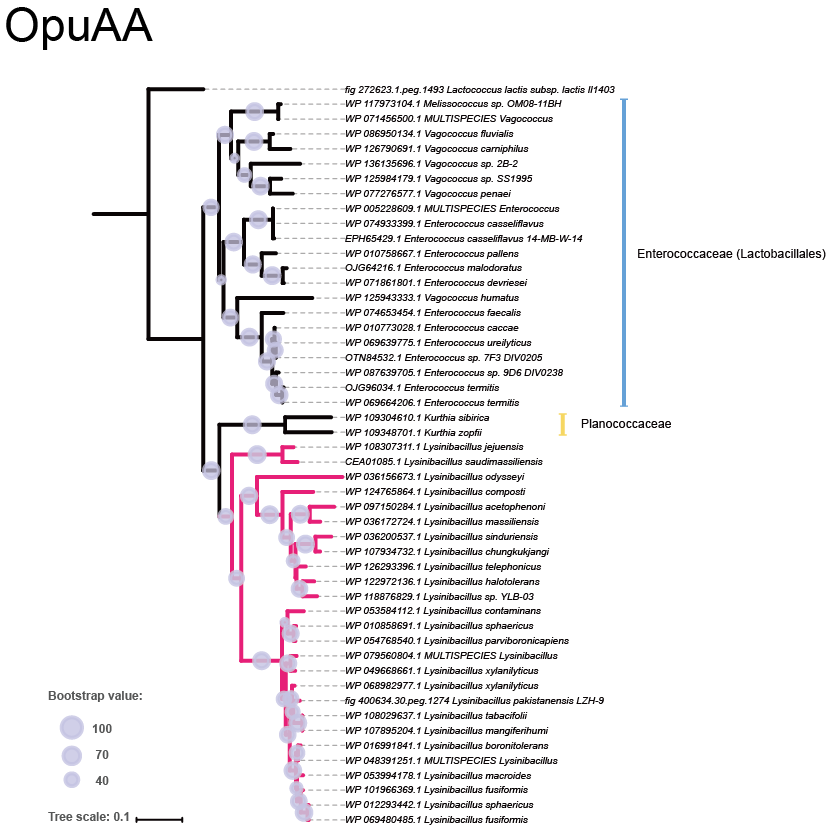


**Figure S31.** Maximum likelihood phylogenetic tree of ATP-binding cassette transporter component OpuAA sequences derived from *Lysinibacillus* spp. strains and other representative species.


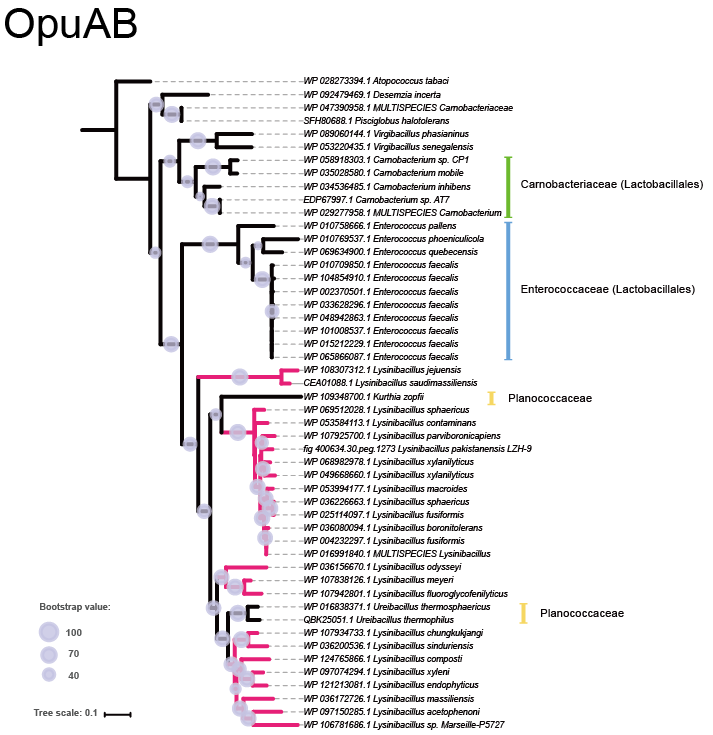


**Figure S32.** Maximum likelihood phylogenetic tree of ATP-binding cassette transporter component OpuAB sequences derived from *Lysinibacillus* spp. strains and other representative species.


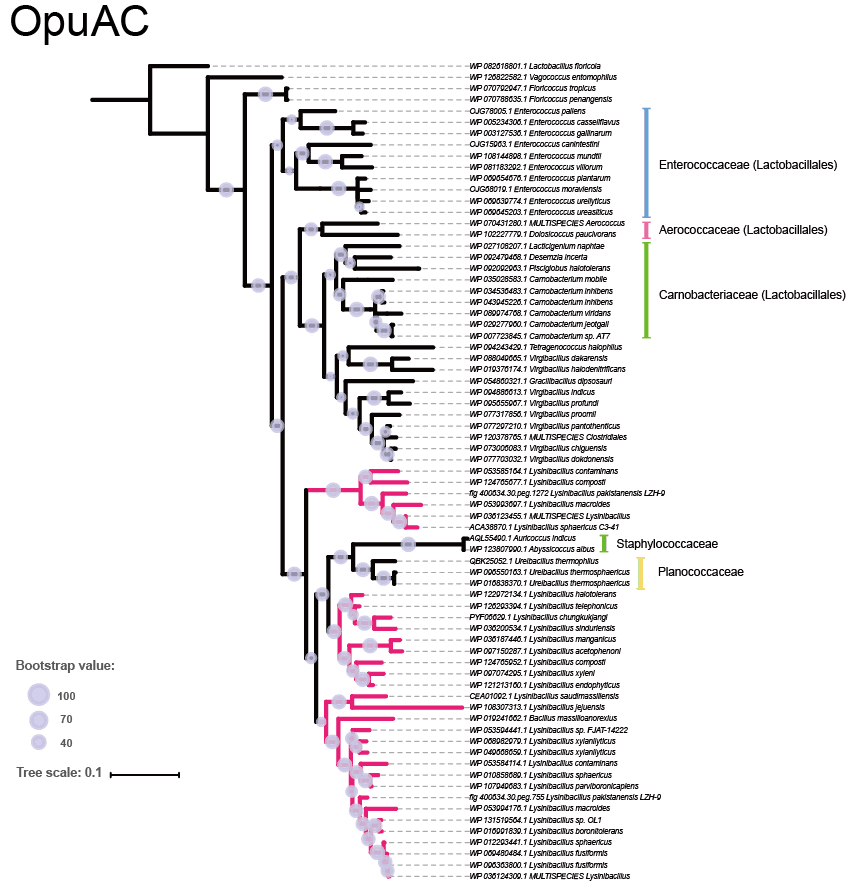


**Figure S33.** Maximum likelihood phylogenetic tree of ATP-binding cassette transporter component OpuAC sequences derived from *Lysinibacillus* spp. strains and other representative species.


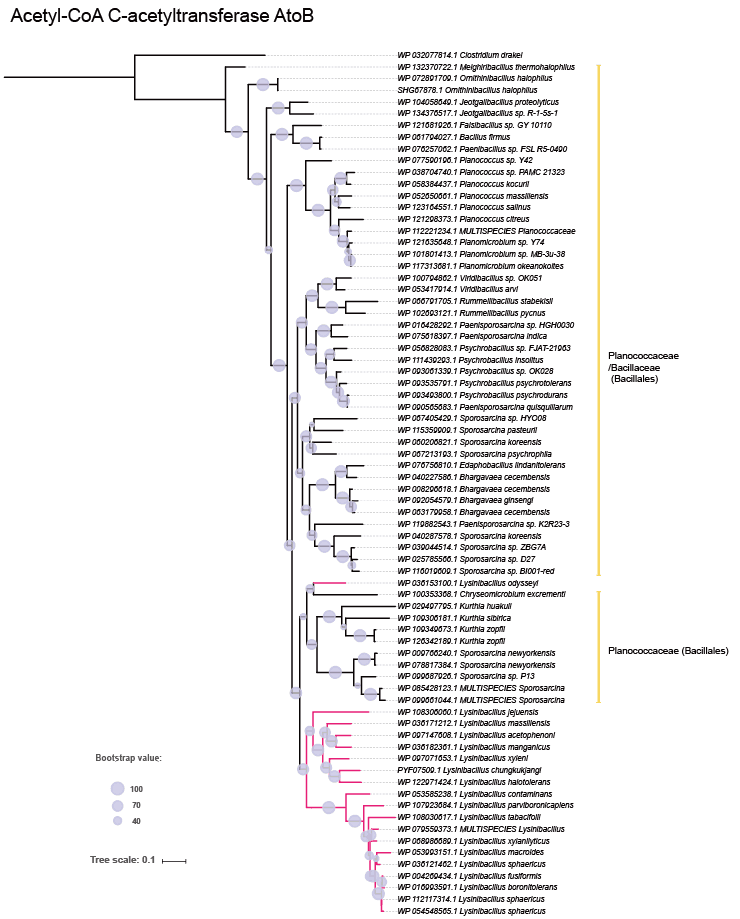


**Figure S34.** Maximum likelihood phylogenetic tree of Acetyl-CoA C-acetyltransferase (AtoB) sequences derived from *Lysinibacillus* spp. strains and other representative species.


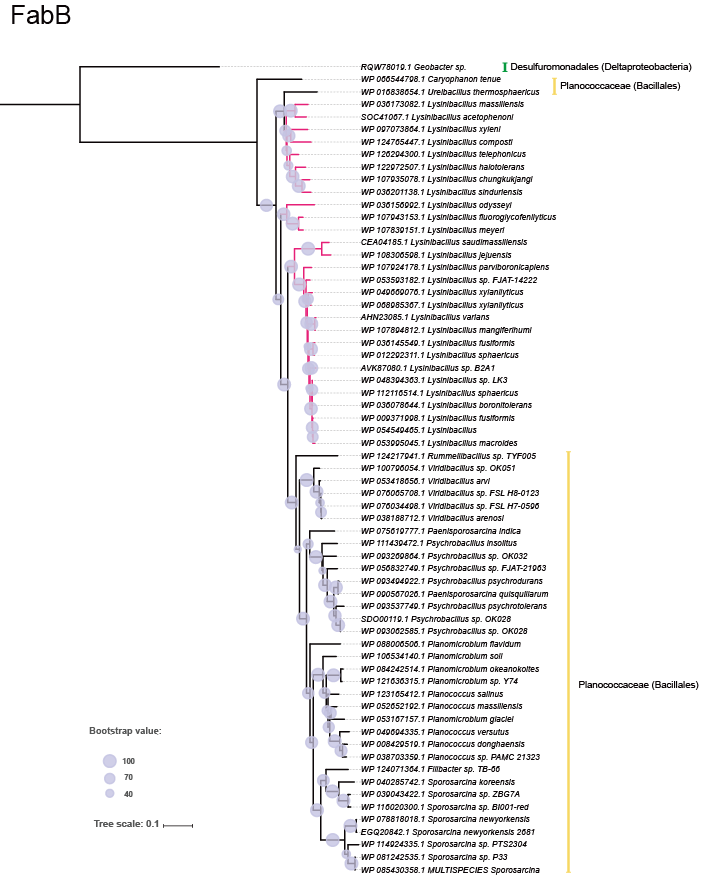


**Figure S35.** Maximum likelihood phylogenetic tree of 3-hydroxybutyryl-CoA dehydrogenase (FadB) sequences derived from *Lysinibacillus* spp. strains and other representative species.


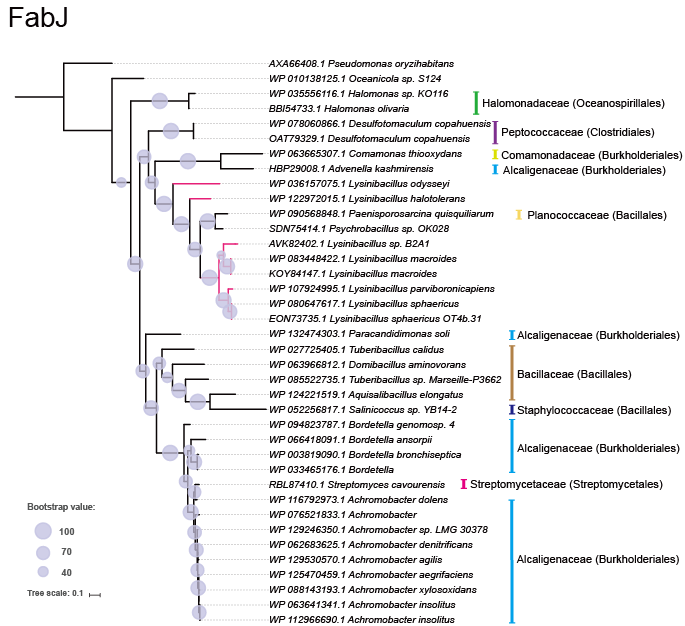


**Figure S36.** Maximum likelihood phylogenetic tree of 3-hydroxyacyl-CoA dehydrogenase/enoyl-CoA hydratase (FadJ) sequences derived from *Lysinibacillus* spp. strains and other representative species.


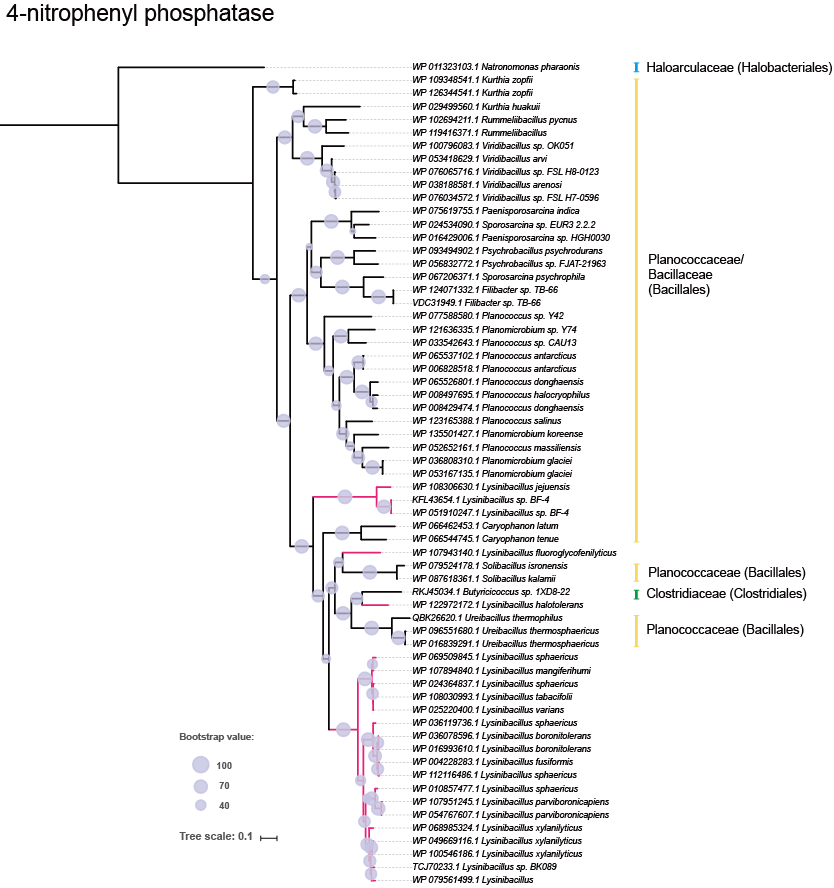


**Figure S37.** Maximum likelihood phylogenetic tree of 4-nitrophenyl phosphatase sequences derived from *Lysinibacillus* spp. strains and other representative species.


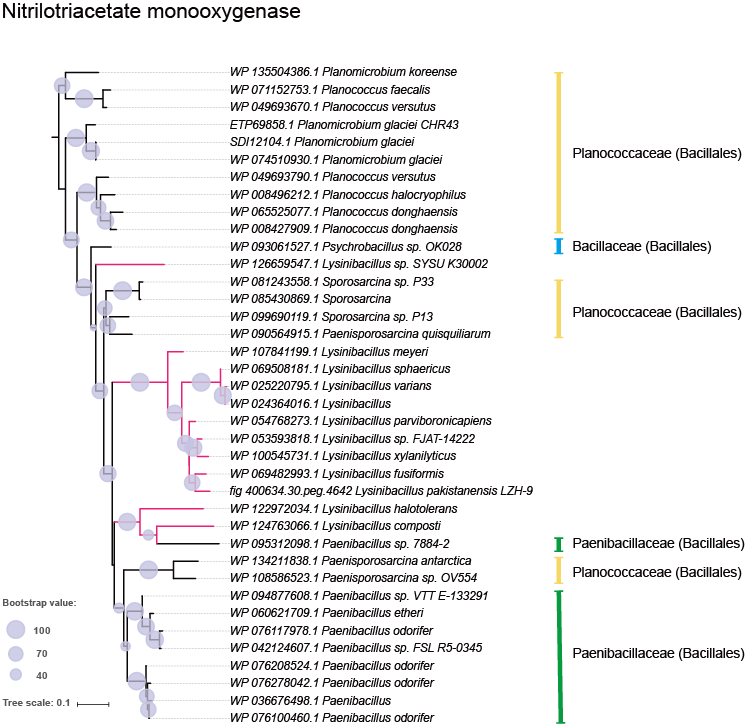


**Figure S38.** Maximum likelihood phylogenetic tree of nitrilotriacetate monooxygenase component B sequences derived from *Lysinibacillus* spp. strains and other representative species.

**
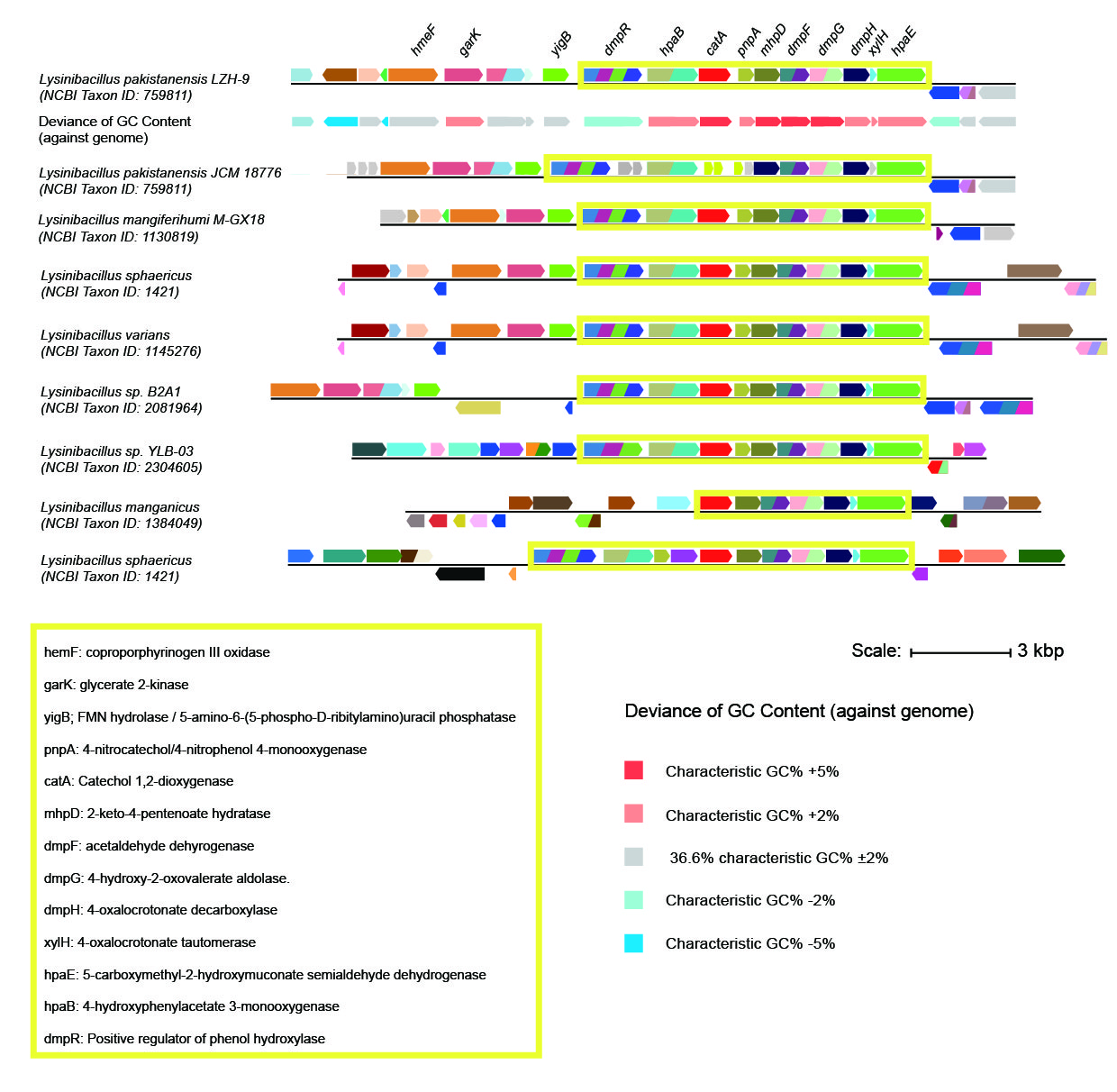
**

**Figure S39.** Synteny analysis of a xenobiotics biodegradation and metabolism related gene cluster *dmpR-hpaB-dmpB-pnpA-mhpD-dmpFGH-xylH-hpaE* derived from *L. pakistanensis* LZH-9, and other representative species in *Lysinibacillus* and GC contents comparison against the genome of *L. pakistanensis* LZH-9.


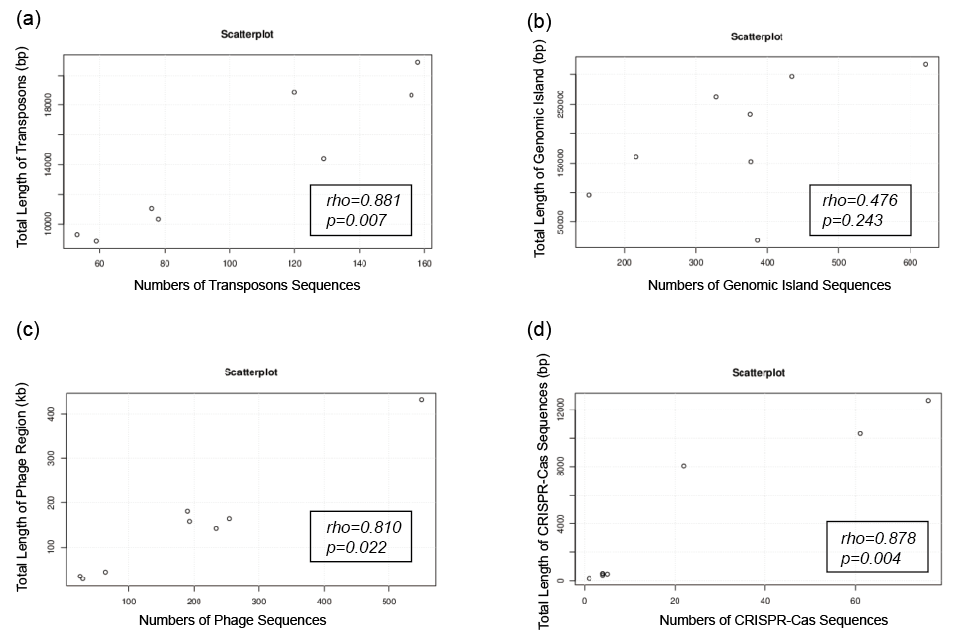


**Figure S40.** Scatterplots of the relationship between: (a) the numbers of transposons sequences and total length of transposons, (b) the numbers of genomic island sequences and total length of genomic island, (c) the numbers of phage sequences and total length of phage region, (d) the numbers of CRISPR-Cas sequences and total length of CRISPR-Cas sequences detected in genomes in table 2.


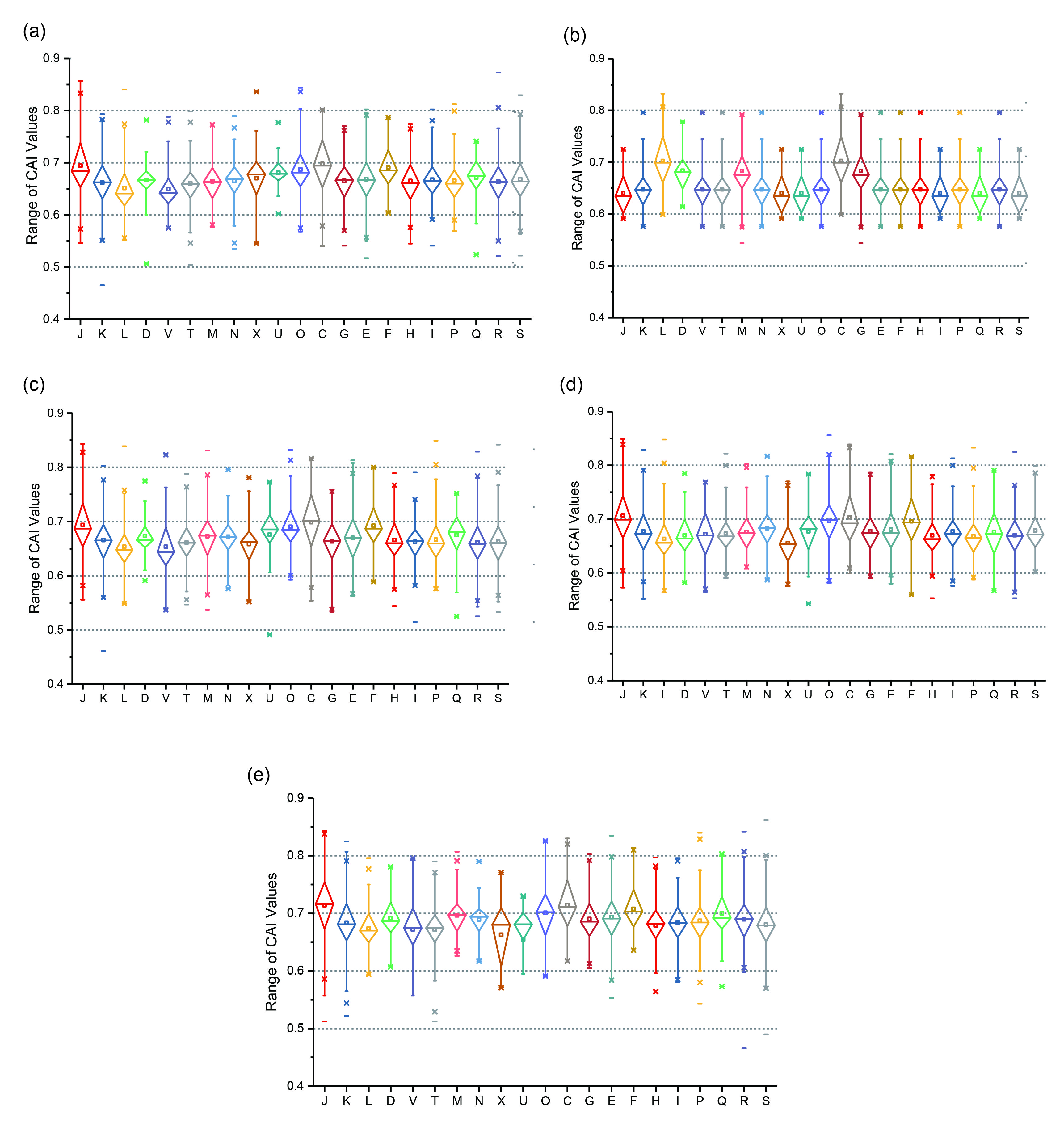


**Figure S41.** Ranges of CAI values of different genes in the genome of five strains belonged *Lysinibacillus* based on COG classification. (a) *L. sphaericus* OT4b.31; (b) *L. contaminans* DSM 25560; (c) *L. parviboronicapiens* VT1065; (d) *L. mangiferihumi* M-GX18*;* (e) *L. xylanilyticus* t26.


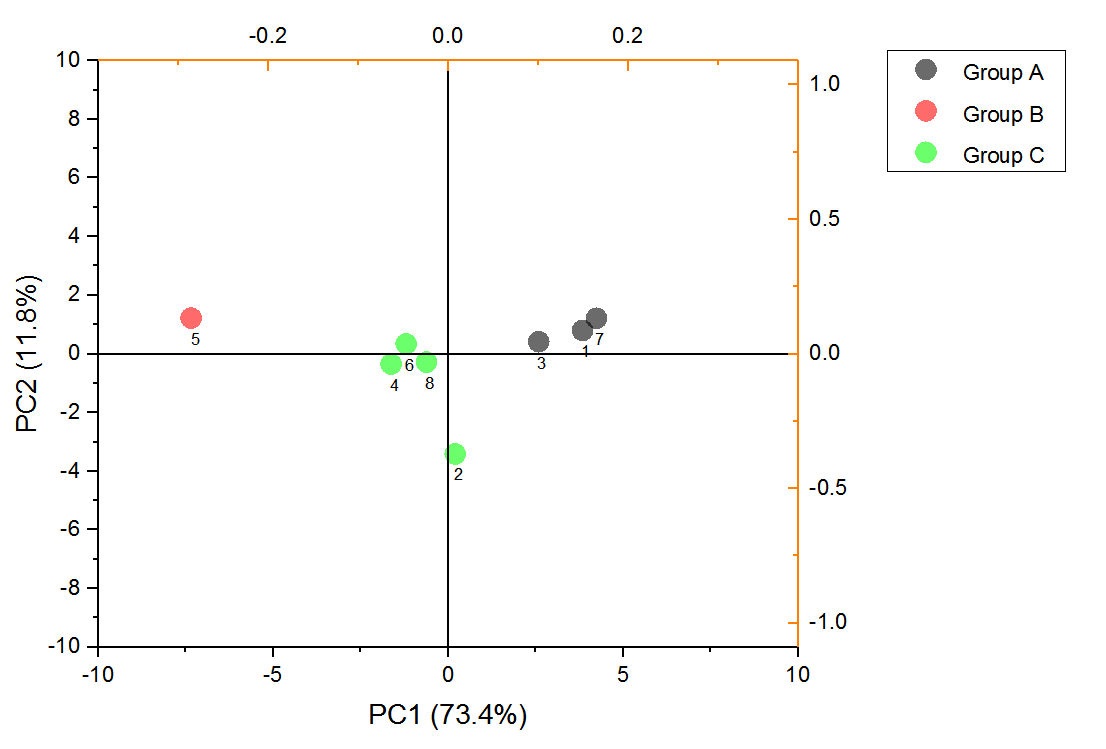


**Figure S42.** Principal coordinates analyses (PCoA) of average CAI values based on COG classes were conducted to visualize the similarity or dissimilarity of expression pattern among different strains of the *Lysinibacillus* (1: *L. sphaericus* OT4b.31, 2: *L. contaminans* DSM 25560, 3: *L. parviboronicapiens* VT1065, 4: *L. mangiferihumi* M-GX18, 5: *L. xylanilyticus* t26, 6: *L. pakistanensis* JCM 18776, 7: *Lysinibacillus* sp. UBA7518, 8: *L. pakistanensis* LZH-9). Result showed strains in this study were clustered into three groups (A, B, C) by PCo2 (accounting for 73.4%) based on COG classes.
